# Uncovering the mechanism of selective stabilization of high-energy diastereoisomers via inclusion

**DOI:** 10.1007/s00214-023-03077-7

**Published:** 2023-12-12

**Authors:** Meagan S. Oakley, Madaline R. Oakes, Brian D. Wagner, Jason K. Pearson

**Affiliations:** 1 Department of Chemistry, The University of Manchester, Oxford Road, Manchester, M13 9PL UK; 2 Chemical Theory Center, Department of Chemistry, Minnesota Supercomputing Institute, University of Minnesota, Minneapolis, MN 55455 USA; 3 Department of Chemistry, University of Prince Edward Island, Charlottetown, PE C1A 4P3 Canada

**Keywords:** Density functional theory, Inclusion, Non-bonded interactions, Selective stability, Stereoisomer, Complex, Copper, Cyclam, Cucurbituril

## Abstract

Supramolecular systems may be used to stabilize otherwise unstable isomers to find alternative synthetic pathways. It has been reported that cucurbit[8]uril can stabilize *trans*-I and *trans*-II Cu$^{{\textrm{II}}}$ cyclam, whereas *trans*-III is the only non-substituted *trans* Cu$^{{\textrm{II}}}$ cyclam diastereoisomer found outside of the host molecule experimentally. Quantum chemistry methods can provide valuable insight into the intermolecular interactions involved in these inclusion complexes. All five possible *trans* diastereoisomers of Cu$^{{\textrm{II}}}$ cyclam were studied within the host molecule to calculate the interaction energy and free energy of association for each complex. The relative free energies of the five free cyclams confirm that *trans*-I and *trans*-II are the most energetically accessible diastereoisomers from the initial *trans*-III starting point. Energy decomposition analysis was used to identify the attractive and repulsive interactions between cyclam and cucurbit[8]uril and showed that *trans*-II encounters repulsive forces almost three times greater than *trans*-I, which may explain the 7:3 ratio of *trans*-I to *trans*-II within cucurbit[8]uril that occurs experimentally. Optimized complex geometries with *trans*-III, IV, and V show that the cyclams protrude out of cucurbit[8]uril, whereas *trans*-I and *trans*-II become more encapsulated and elongate the host, suggesting that the position of the cyclam is extremely important when forming non-covalent interactions. Our results agree with the experimental findings and provide greater insight into why the most stable isolated cyclam diastereoisomer, *trans*-III, does not form a complex.

**Supplementary Information:**

The online version contains supplementary material available at 10.1007/s00214-023-03077-7.

## Introduction

Host-guest inclusion complexes have a wealth of applications from drug capture for targeted delivery [1], to selective binding for molecule detection through molecular sensor design [2]. Such inclusion complexes are primarily stabilized through non-covalent interactions between the host and guest such as van der Waals forces, hydrogen bonding and the hydrophobic effect [3]. This added stability can be exploited to preferentially explore otherwise inaccessible chemistry.

For example, it has been shown that cucurbituril molecules [4] have the ability to act as nanoreactors [5] by stabilizing high energy compounds and providing alternate reaction pathways for chemical synthesis. Cucurbit[*n*]urils (CB*n*) are named for their pumpkin-like shape (pumpkin, from the cucurbitaceae family) and are made up of *n* repeating glycouril units joined by pairs of methylene bridges, with *n* usually varying from 5 to 8. Cucurbiturils are generally rigid host molecules with carbonyl groups lining the cavity, contributing to the highly negative electrostatic potential within the host. Preferred guests are therefore mainly neutral or cationic [6].

**Fig. 1:**
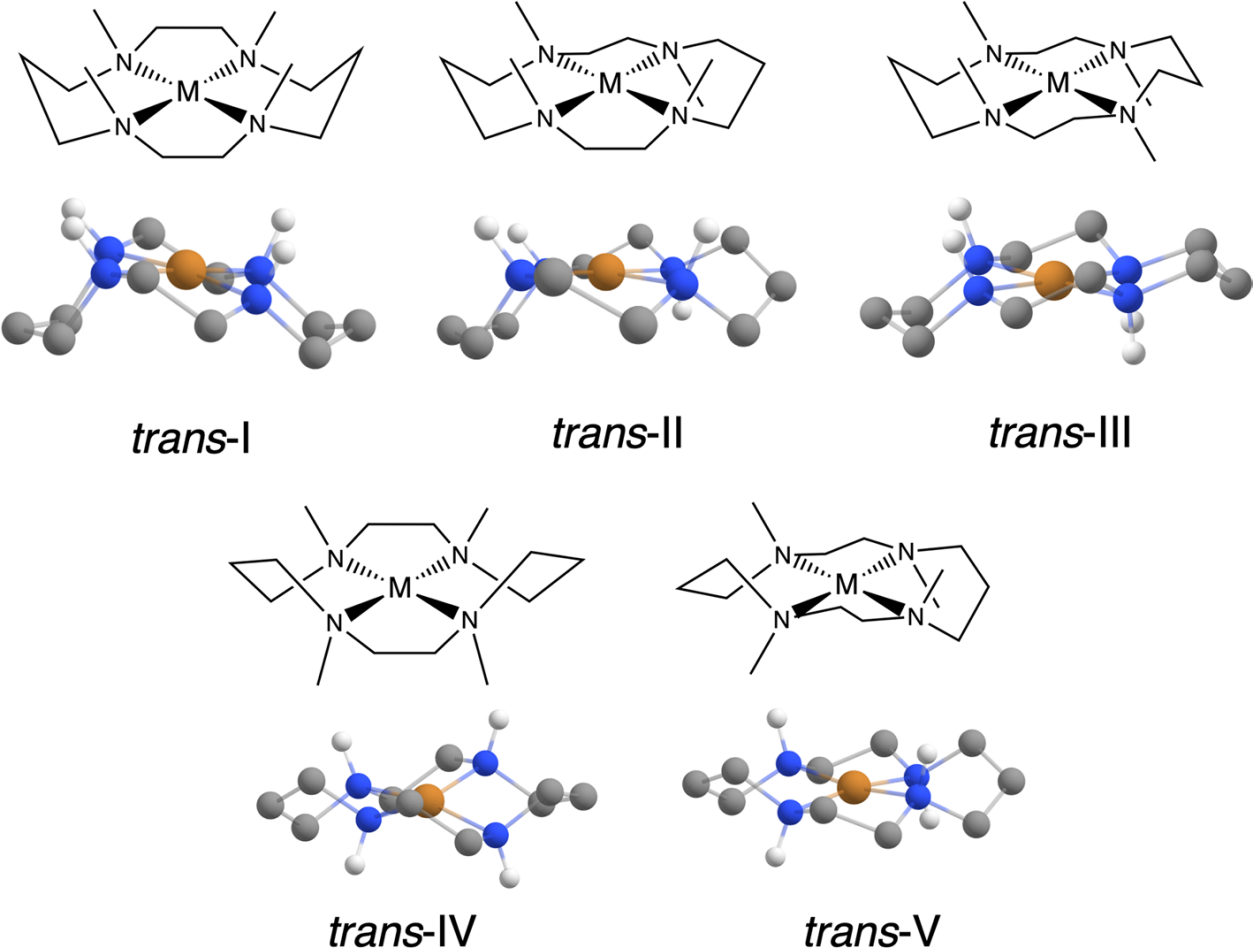
The possible *trans* diastereoisomers a metal-coordinated cyclam can possess: *trans*-I through *trans*-V

In 2009, Hart et al. [7] reported the first stabilization of non-substituted *trans*-I and *trans*-II Cu$^{\textrm{II}}$ cyclam diastereoisomers by inclusion within a CB8 host molecule. Cyclam, or 1,4,8,11-teraazacyclotetradecane, is a 14 membered macrocycle which can bind to a variety of metals (such as Cu, Zn, Ni, Cr, Tc, and Ru) and is used in many clinical applications such as radionuclides for diagnostic imaging and stem cell mobilization [8]. Cyclam complexes can generally be observed in one of five diastereoisomers, as shown in Fig. 1.

However, only the *trans*-III diastereoisomers has been observed in its free, uncomplexed form, owing to the fact that steric hindrance is minimized [9]. Interestingly, the *trans*-III Cu$^{\textrm{II}}$ cyclam complex was *not found* within the CB8 cavity. It was proposed that hydrogen bonding between the CB8 host and the Cu$^{\textrm{II}}$ cyclam guest was accountable for stabilizing the unexpected high-energy cyclams. However, it is important to point out that similar studies with Ni$^{\textrm{II}}$ cyclam included within CB8 found that *only* the *trans*-III cyclams were isolated [7, 10]. In this work we systematically explore, *in silico*, the inclusion chemistry of Cu$^{\textrm{II}}$ cyclam with CB8. By using quantum chemistry methods, we seek to rationalize the surprising experimentally observed stability of higher energy Cu$^{\textrm{II}}$ cyclam structures within CB8.

## Computational methods

**Fig. 2:**
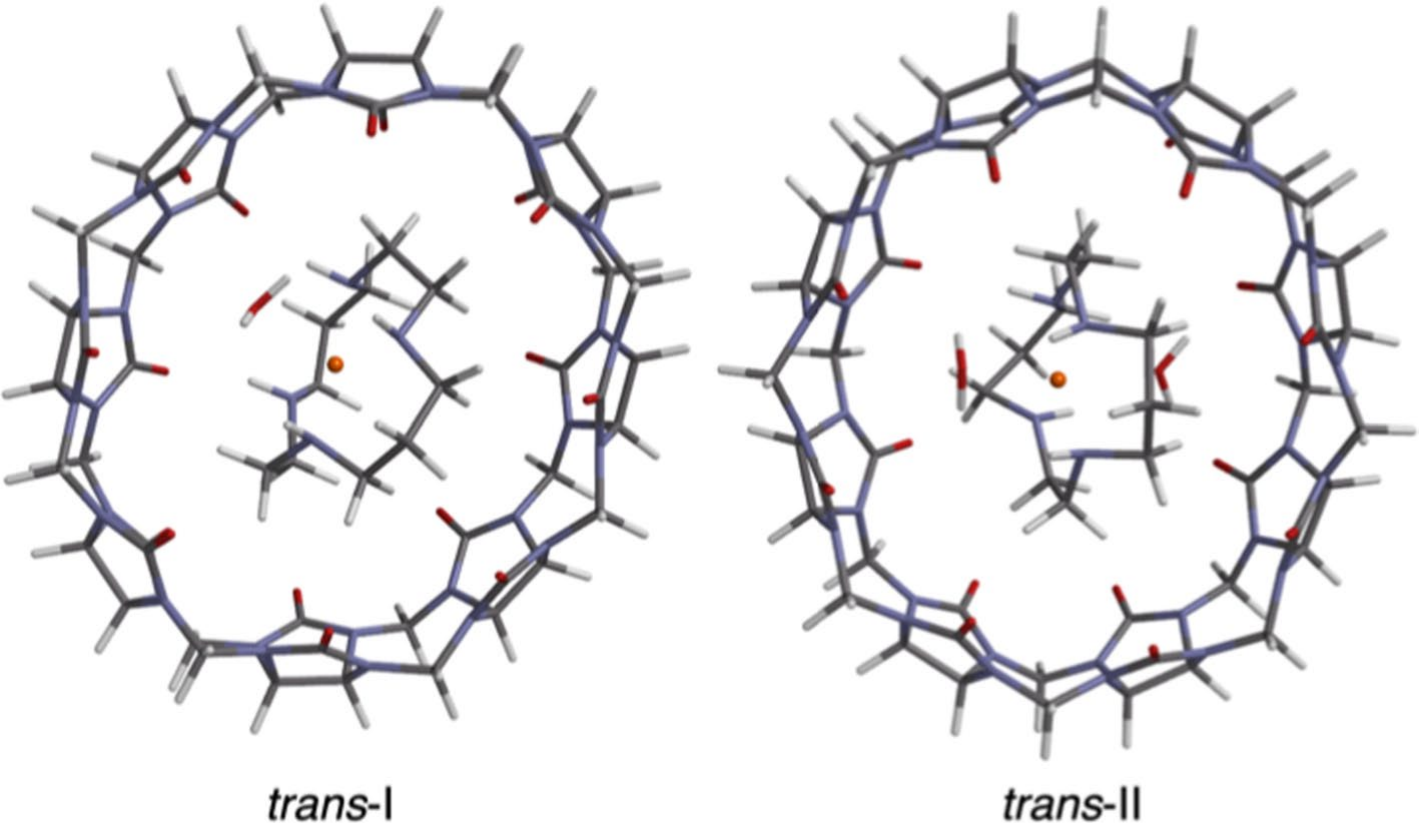
Crystal structures of *trans*-I (right) and *trans*-II (left) Cu$^{\textrm{II}}$ cyclam within cucurbit[8]uril, with one and two waters coordinated to the copper centre, respectively

To determine the relative stability of cyclams within and outside CB8 under a variety of conditions, geometry optimizations were performed on each of the five Cu$^{\textrm{II}}$ cyclam diastereoisomers as free molecules, complexed to 1 and 2 water solvent molecules, and within CB8. The crystal structures obtained by Hart et al. [7] for the *trans*-I and *trans*-II Cu$^{\textrm{II}}$ cyclams exhibit 1 and 2 water molecules, respectively, coordinated to the copper centre of the cyclam (see Fig. 2). As such, it is important to explore the extent to which such microsolvation plays a role in the relative thermodynamics of this system, so all geometry optimizations were performed in both the gas phase and with an implicit water solvation model based on density (SMD) [11].

All structures were optimized with density functional theory (DFT) and frequency calculations were performed on each of the single point structures to ensure that they corresponded to a minimum on the potential energy surface. To do this, we used the TPSS [12] functional paired with the def2-TZVP [13, 14] basis set and the D3 empirical dispersion correction with Becke-Johnson damping for appropriate description of dispersion interactions [15]. All optimizations, frequency and single-point energy calculations were performed with the Gaussian 16 (Rev B.01) program [16] and tight convergence criteria.

For a more detailed analysis of intermolecular and intramolecular interactions, an extended transition state energy decomposition with natural orbitals for chemical valence [17, 18] (ETS-NOCV) analysis was performed on the crystal structures to quantify the magnitude of attractive and repulsive forces between cyclam and CB8. This energy decomposition analysis (EDA) was done with the BLYP [19, 20] density functional incorporating the D3 dispersion correction [21] and the triple-$\zeta$ polarized TZ2P basis set [22] with the Amsterdam Density Functional 2016 program [23–25]. This computational approach has been shown previously to be accurate for non-covalent interactions [26].

The quantum theory of atoms in molecules [27] (QTAIM) was used to compute bond critical points between cyclam and CB8 to determine the presence and relative strength of individual interactions within the AIMAll package [28] (Version 14.04.17). Additionally, non-covalent interactions within the complexes were predicted and visualized with the NCIplot [29, 30] program through an analysis of the electron density, $\rho$, and the reduced gradient density, $s(\rho )$. The isosurfaces are generated using a $\rho$ < 0.07 *a.u.* cutoff to capture mainly weak interactions.

## Results and discussion

### Free *trans*-X Cu$^{\textrm{II}}$ cyclam

**Fig. 3:**
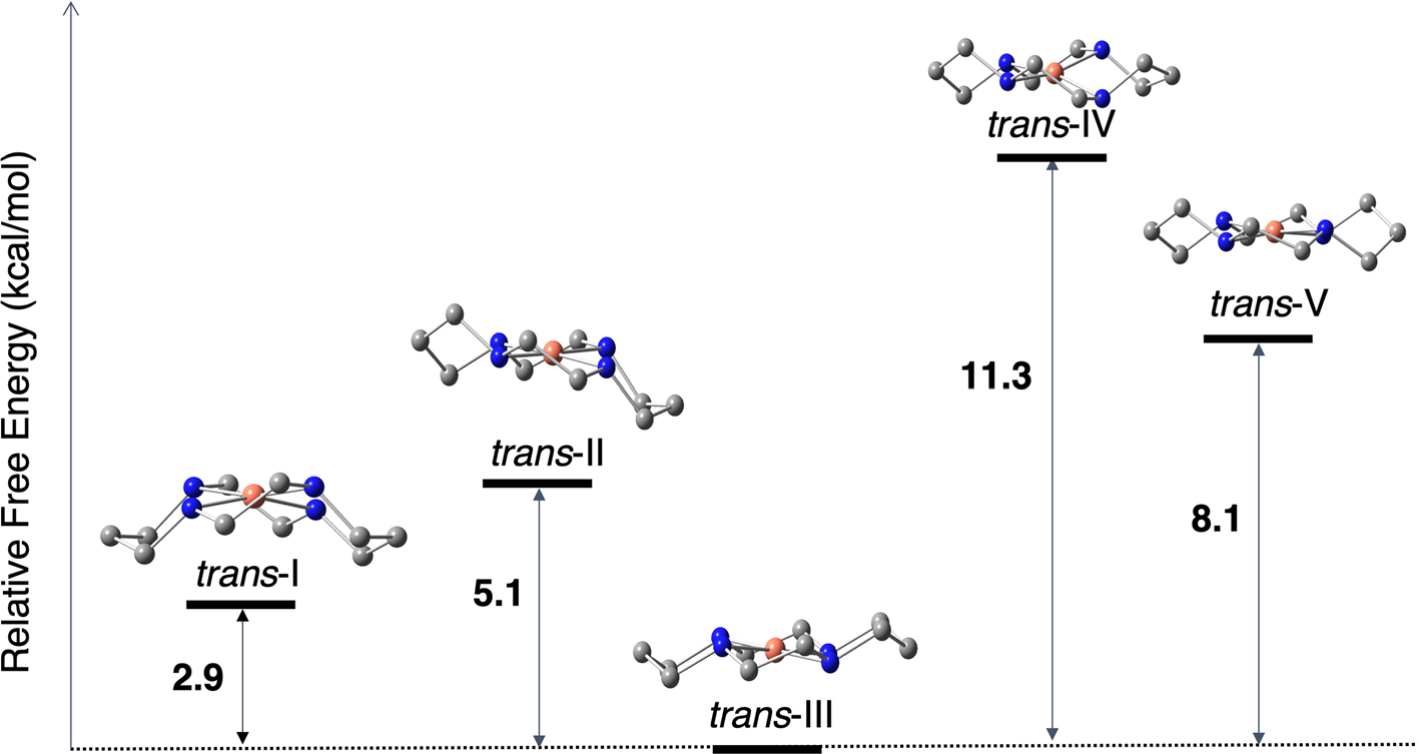
The relative total Gibbs free energies (in kcal/mol) of the five *trans* diastereoisomers of Cu$^{\textrm{II}}$ cyclam. The free energies are computed with implicit solvation

A comparison of the relative total Gibbs free energies of each diastereoisomer of *trans* Cu$^{\textrm{II}}$ cyclam is shown in Fig. 3. These free energies are computed with TPSS/def2TZVP with implicit water solvent taken into account with SMD. The results agree with experiment in that the most stable diastereoisomer of the free uncomplexed Cu$^{\textrm{II}}$ cyclam is *trans*-III [9].

The difference in free energy between *trans*-III to *trans*-I is the smallest at 2.9 kcal/mol, and *trans*-II is the next closest at 5.1 kcal/mol higher. Furthermore, the experimental cyclam@CB8 complex is observed in a 7:3 ratio of *trans*-I and *trans*-II included in CB8, respectively, and this is somewhat supported by the free energy differences in that *trans*-I would be slightly more energetically accessible than *trans*-II. The population probability of the free cyclams were calculated via a Boltzmann distribution at 298 K: as expected *trans*-III has a >99% probability, whereas *trans*-I and *trans*-II are less likely to be populated at 0.75% and 0.02%, but shows that it is an order of magnitude more likely that *trans*-I is populated with respect to *trans*-II.

The *trans*-IV and *trans*-V cyclams are higher in free energy (11.3 kcal/mol for *trans*-IV and 8.1 kcal/mol for *trans*-V) with respect to the most stable cyclam; at 298 K, *trans*-IV and *trans*-V are not populated. This may explain why these diastereoisomers are not experimentally observed within CB8.

When considering the thermodynamic pathway for inclusion, it is instructive to next look at the activation strain required to distort the free cyclam isomers to their optimized geometry within the CB8 cavity. That is, we first optimize the structure of the complexed cyclam within CB8 and then compare this energy (of just the cyclam) to its free analogue, and the results are reported in Table 1 in both gas phase and implicit water solvent.

**Table 1:** Strain energy (in kcal/mol) required to distort the geometry of free *trans* Cu$^{{\textrm{II}}}$ cyclam to the structure included in cucurbit[8]uril computed with TPSS/def2TZVP

System	Gas phase	Solvated
*trans*-I $\rightarrow$ *trans*-I@CB8	0.4	0.2
*trans*-II $\rightarrow$ *trans*-II@CB8	1.4	0.3
*trans*-III $\rightarrow$ *trans*-III@CB8	1.8	0.4
*trans*-IV $\rightarrow$ *trans*-IV@CB8	1.0	0.2
*trans*-V $\rightarrow$ *trans*-V@CB8	0.7	0.5

The strain energies of each diastereoisomer are all within approximately 1.8 kcal/mol, which shows that the strain of inclusion may not be the limiting step in this multi-faceted process. It is also important to note here that the computationally optimized structures of the *trans*-I and *trans*-II cyclams included within CB8 differ somewhat from the reported experimental crystal structures (see Sect. 3.2.1). This is not surprising owing to the lack of a condensed phase within our models, but it does afford us the opportunity for comparison. For the implicit solvation calculations, there is minimal difference in energetics as all are within at most 0.5 kcal/mol. Together with the data in Fig. 3, it may be more likely that *trans*-III cyclam structurally changes to *trans*-I or *trans*-II cyclam in order to be favourably included in the CB8 host.

**Table 2:** Strain energy (in kcal/mol) required to distort the geometry of free *trans*-III Cu$^{{\textrm{II}}}$(cyclam) to each possible *trans* structure included in cucurbit[8]uril computed with TPSS/def2TZVP

System	Gas phase	Solvated
*trans*-III $\rightarrow$ *trans*-I@CB8	2.1	1.1
*trans*-III $\rightarrow$ *trans*-II@CB8	6.0	4.0
*trans*-III $\rightarrow$ *trans*-III@CB8	1.8	0.4
*trans*-III $\rightarrow$ *trans*-IV@CB8	12.0	10.1
*trans*-III $\rightarrow$ *trans*-V@CB8	7.8	6.8

Table 2 reports the strain energy required for *trans*-III cyclam to convert into each of the five diastereoisomers studied here. Unsurprisingly, the process in which *trans*-III distorts to include itself within CB8 is the lowest energy at 1.8 kcal/mol, however, this does not explain the absence of this complex in experiment. The energetics involved in distorting *trans*-III to *trans*-I@CB8 and *trans*-II@CB8 requires 2.1 kcal/mol and 6.0 kcal/mol, respectively, which also agrees with the 7:3 ratio of *trans*-I@CB8 to *trans*-II@CB8, as observed experimentally. The strain energy is higher for *trans*-III to distort into *trans*-IV@CB8 and *trans*-V@CB8 compared to all other diastereoisomers, and when one takes into consideration that, in the free state, the *trans*-IV and *trans*-V cyclams are much less accessible in solution, it becomes clear as to why these stereoisomers are not observed. The energetic trends are similar when using results computed using implicit solvation, although the relative energies are slightly lower.

We also studied whether copper-solvent coordination, as seen in the experimental crystal structures, had an effect on stabilization of the stereoisomers and the results are reported in Table S1. Similar relative energy trends to those compounds with no water coordinated are seen for both one and two-water coordinated structures.

These results provide an energetic explanation as to why the CB8 host favours the *trans*-I and *trans*-II cyclam conformations, and in particular the *trans*-I cyclam conformation (in good agreement with experiment). This seems to be at least due to cavity shape since, at this point, we have focussed solely on geometric distortions of the various cyclam stereoisomers. However, it is not yet clear why *trans*-III is not stabilized within the host molecule, so we turn to modelling the full complex for more understanding.

### *trans* Cu$^{\textrm{II}}$ Cyclam@Cucurbit[8]uril crystal structures

**Fig. 4:**
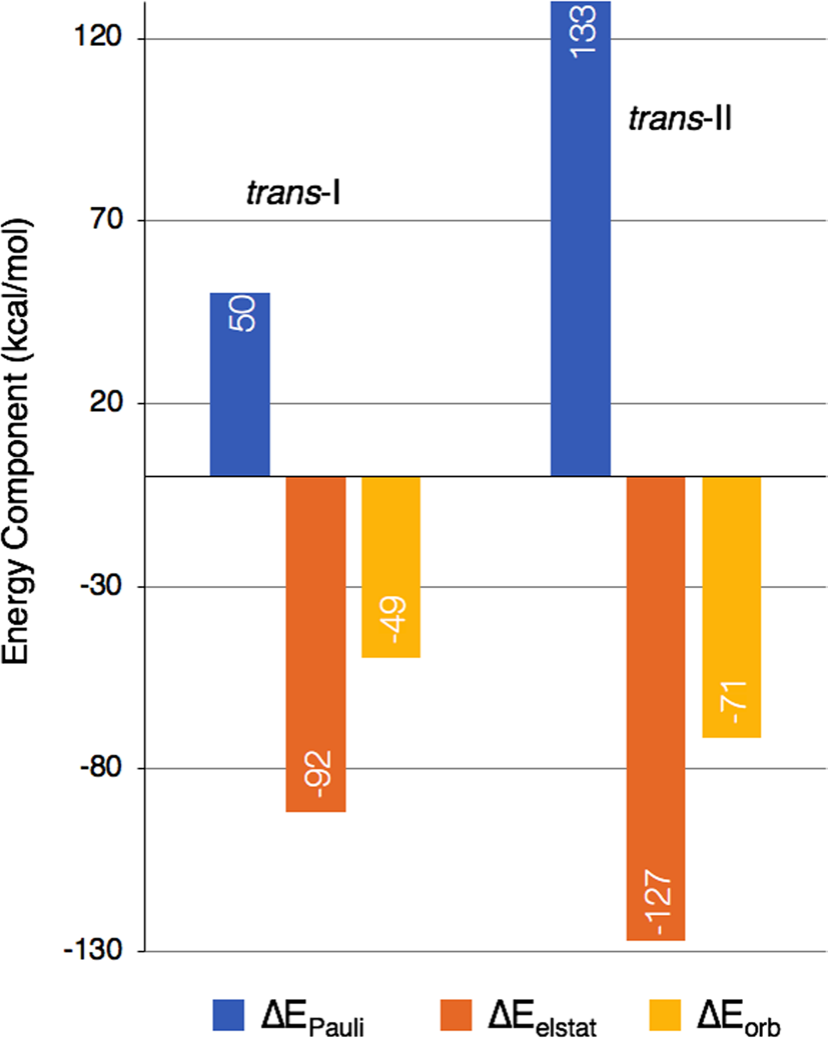
Energy decomposition analysis components for the *trans*-I (left) and *trans*-II (right) crystal structures (reported in kcal/mol)

First, we study the experimental crystal structures, which are used here to better understand the interactions between cyclam and CB8 [7]. Energy decomposition analysis was used to determine specific contributions of the interactions between the host and guest molecules. This method considers energetics of molecular fragments alone and the full interacting complex in order to decompose the interaction energy into attractive and repulsive contributions according to:

1 $$\begin{aligned} \Delta E_{int}=\Delta E_\textrm{Pauli}+\Delta E_\textrm{elstat}+\Delta E_\textrm{orb} \end{aligned}$$

where the first term accounts for Pauli repulsion and the second and third terms account for electrostatic and orbital interaction, the attractive forces. The EDA values are reported in Fig. 4 for both crystal structure complexes. Here, the complex is partitioned into two molecular fragments: CB8 alone (host fragment) and cyclam with coordinated water solvent (guest fragment) to account for the interaction between the host and guest molecules.

Each complex is stable, so the repulsive forces must be overcome by the stronger attractive forces. The Pauli repulsion components for each complex are quite different; the value for *trans*-I is about one-third of the value for *trans*-II. This may also contribute to the 7:3 ratio of the complexes found experimentally—there is simply more repulsion involved in forming the *trans*-II complex. Additionally, we must also consider that *trans*-II has one extra water molecule captured inside CB8, which could be the source of the extra repulsion. Since *trans*-II has only ever been isolated with two water molecules experimentally, we can deduce that the second water molecule may be required to stabilize this stereoisomer inside CB8.

The crystal structures offer a path to understanding why these specific diastereoisomers are stabilized. However, we must turn to quantum chemistry methods to determine why the other diastereoisomers are not found, especially in the case of *trans*-III.

#### *trans* Cu$^{\textrm{II}}$ Cyclam@Cucurbit[8]uril computed structures

**Fig. 5:**
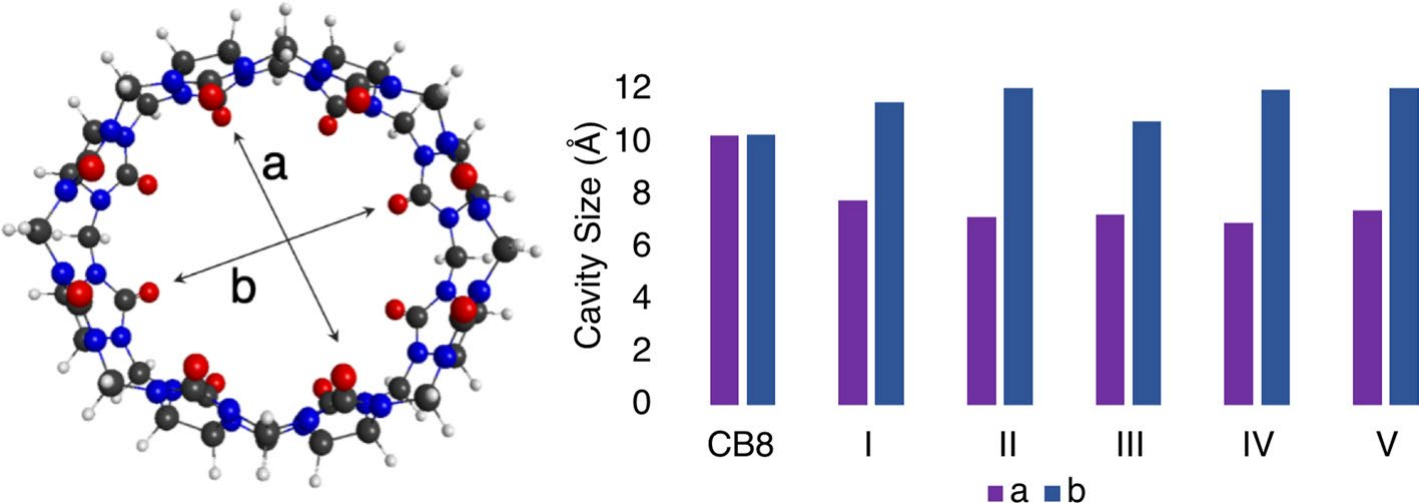
Left: The length (*a*) and width (*b*) dimensions of the CB8 cavity. Right: *a* and *b* values (in Å) for the free CB8 and each of the five possible complexes

The geometries of all five complexes were optimized at the TPSS/def2-TZVP level of theory. The dimensions of the CB8 cavity were studied as a probe into the geometric effects of inclusion. The cavity dimensions (length, *a*, and width, *b*) were measured for each computed structure and are reported in Fig. 5.

**Fig. 6:**
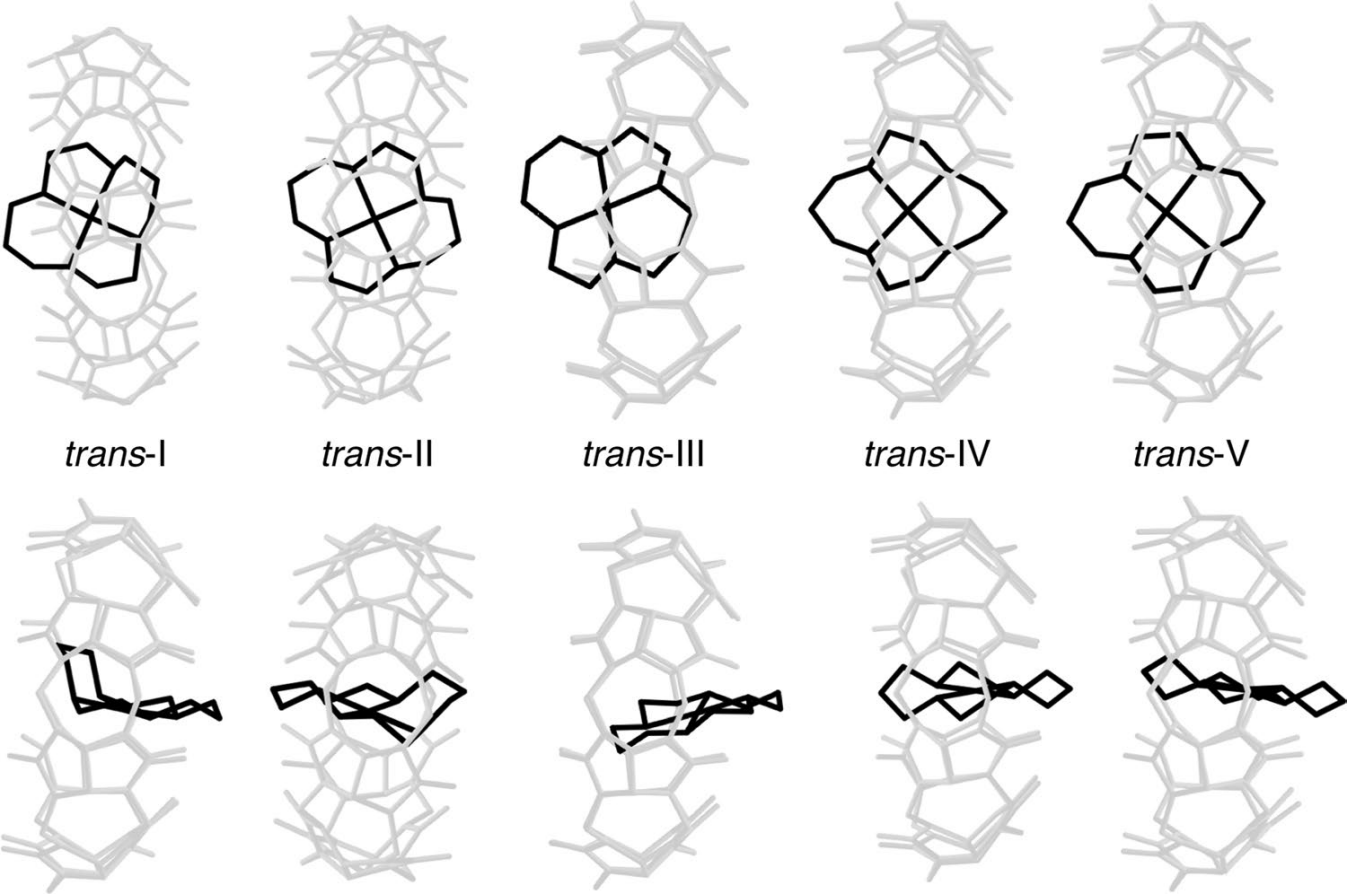
The positions of *trans* Cu$^{{\textrm{II}}}$ cyclam (black) inside cucurbit[8]uril (grey) for each of the *trans* diastereoisomers considered. Hydrogens are hidden for clarity

The *a* parameter is defined as the smallest distance between opposite carbonyl oxygen atoms (4 glycouril units away) lining the cavity. Since the structure is symmetric, the *b* parameter is defined as the largest distance between opposing carbonyl oxygen atoms, or perpendicular to the *a* parameter. As a reference point, the free CB8 host was measured and found that the *a* and *b* values are similar as the cavity is circular. However, when any cyclam is included, the CB8 elongates to make space for the guest molecule. Interestingly, the CB8 cavity becomes more oval-shaped for those structures which are isolated experimentally, but the *trans*-III diastereoisomers has less of an impact on the shape of the cavity, which may be why it is not observed. In comparison, the cavity dimensions for the crystal structures are *a* = 8.39 Å and *b* = 11.32 Å which is slightly rounder than what is shown for the computed complexes. This is to be expected considering the crystal packing may influence the cavity structure. Additionally, the optimized geometries for *trans*-I and *trans*-II differ somewhat than the other diastereoisomers, in that they are almost fully encapsulated inside CB8. This may be contrasted with the other cyclam isomers, which are rotated slightly so that they are diagonal or protrude out of the cavity. Figure 6 shows the positions of each diastereoisomers inside CB8. Since *trans*-I and *trans*-II are bent in the direction of the arc in the cavity, stronger and closer interactions with the host molecule are possible.

The structural parameters of the included Cu$^{{\textrm{II}}}$ cyclams are reported in Table S3 in the supporting information, where we are able to compare our computed models of *trans*-I and *trans*-II to the observed crystal structures. It is found that the geometries are quite similar, which is likely due to the rigidity of the first coordination sphere of Cu.

The quantum theory of atoms in molecules [27] (QTAIM) provides a quantitative analysis of the electron density and deeper insight into the nature of the interactions within the complex. This model locates bond critical points (BCP) which correspond to the point of minimum electron density between two atoms, or nuclear basins. The value of the electron density, $\rho$, at the BCP tends to correlate to the strength of the interaction. We have therefore performed an analysis of the electron density at the bond critical points between the hydrogen atoms on the cyclam and the oxygen atoms on the CB8. The number of interactions, ($n_{int}$), does not seem to correlate with the strength of interaction, however, an average approach was also taken to analyze the magnitude of the electron density at the BCPs which allows for comparison between compounds with varying numbers of interactions. The full electron density analysis for the H-O interactions is reported in Tables S2-S6 in the supporting information.

**Table 3:** The number of non-covalent interactions (*n*$_{int}$), the sum of and average electron density values at the bond critical point ($\rho _{BCP}$) and the Laplacian of the electron density at bond critical points ($\nabla ^2\rho _{BCP}$) between hydrogen atoms on Cu$^{\textrm{II}}$ cyclams and oxygen atoms on cucurbit[8]uril, reported in atomic units

		Sum of	Average	Average
System	*n*$_{int}$	$\rho _{BCP}$ (a.u.)	$\rho _{BCP}$ (a.u.)	$\nabla ^2\rho _{BCP}$ (a.u.)
*trans*-I	18	0.137	0.007	0.029
*trans*-II	19	0.130	0.006	0.026
*trans*-III	16	0.139	0.008	0.033
*trans*-IV	20	0.138	0.007	0.026
*trans*-V	17	0.133	0.008	0.030

Based on the results in Table 3, *trans*-III is the system with the highest electron density at these BCPs on average. This is interesting considering the analogous nickel cyclam complex is the most stable diastereoisomer within CB8. Of the remaining complexes, the ordering of the average $\rho _{\textrm{BCP}}$ approximately follows that of the experimental results although there are very small differences in each case.

**Fig. 7:**
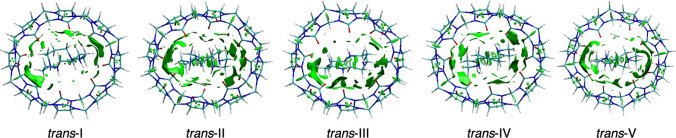
Non-covalent interaction isosurfaces (green) for each computed structure of the Cu$^{\textrm{II}}$ cyclam@CB8 inclusion complex

The NCIplot program [29, 30] utilizes the electron density and allows one to visualize interactions between the host and guest molecules. Isosurfaces representing weak interactions are produced at density critical points where the electron density and the reduced density gradient approach zero. Figure 7 shows each of the five complexes with the weak interactions represented by the green isosurfaces.

The *trans*-I complex has two surfaces on each side of the cyclam which interact with CB8 and another large isosurface on the top side of the complex. The bottom side has a few small surfaces which map to hydrogen bonds between hydrogens on the cyclam and the carbonyl groups lining the cavity. Since the cyclam is shifted to one side, there are considerably less interactions underneath. The *trans*-II complex seems to have slightly larger isosurfaces than *trans*-I, which is also reflected in the number of interactions but the sum of $\rho _{BCP}$ and the average $\rho _{BCP}$ are lower, as reported in Table 3. This shows that while the *trans*-II complex has more interactions, they are slightly weaker than that of *trans*-I. The *trans*-III complex has less interactions on one side than *trans*-I and *trans*-II, and the isosurface is comparable to that of *trans*-V. The *trans*-IV structure is similar to that of *trans*-II, with the interactions being dispersed evenly around the cyclam, whereas the *trans*-V structure is similar to *trans*-I and *trans*-III, which all have some concentration of interactions near the sides of the cyclam and near the top. The side of the cyclam that is further away from CB8 has fewer non-covalent interactions, as expected.

The computed structures were also used to calculate the interaction energy and free energy of association between the host and guest molecules. The free energy of association, $\Delta G\mathrm {_a}$, of each complex was computed using the approach outlined by Grimme [31],

2 $$\begin{aligned} \Delta G_a = \Delta E + \Delta G_{RRHO} + \Delta \delta G_{solv} \end{aligned}$$

where the interaction energy, $\Delta E$, and the thermal corrections, $\Delta G_{RRHO}$ and $\Delta \delta G_{solv}$, are computed with DFT, namely the TPSS functional, in the gas phase and in water solvent, respectively. The low-lying vibrational frequencies ($\omega$ $_0$ $\le$ 100 cm$^{-1}$) are treated within a rigid-rotor model [31] and water solvent effects were accounted for using SMD. Each term in the free energy of association formula is evaluated as the total energies of the individual host and guest components subtracted from the total energy of the complex,

3 $$\begin{aligned} E(trans\text{- }{\textrm{X}}@CB8) - \left[ E(CB8) + E(trans\text{- }{\textrm{III}})\right] \end{aligned}$$

where the $E(trans\text{- }{\textrm{X}}@CB8)$ term corresponds to the energy of the complex of interest. The interaction energy ($\Delta E$), solvation free energy ($\Delta \delta G_{solv}$), and free energy of association ($\Delta G_a$) are reported in Table 4.

The *trans*-IV@CB8 and *trans*-V@CB8 complexes exhibit the lowest interaction energies, $\Delta \textrm{E}$(TPSS), however, it would not be likely that these complexes are formed considering the free cyclams have zero population at 298 K. This leaves only the *trans*-I, *trans*-II, and *trans*-III cyclams, and respective complexes with CB8, thermodynamically available. The *trans*-I@CB8 and *trans*-II@CB8 complexes have similar interaction energy values, which is in agreement with the experimental findings. The calculated complexes do not have water coordinated to copper, so the increased Pauli repulsion felt by *trans*-II may reduce the interaction energy to below that of *trans*-I if explicit solvent was included here.

**Table 4:** The gas-phase interaction energies, solvation free energies, and free energies of association in kcal/mol reported for each stereoisomer complex

Complex	$\Delta \textrm{E}$(TPSS)	$\Delta \delta G_{solv}$	$\Delta G_{a}$
*trans*-I@CB8	$-$126.3	87.0	$-$12.4
*trans*-II@CB8	$-$124.8	82.9	$-$15.5
*trans*-III@CB8	$-$128.0	92.8	$-$8.8
*trans*-IV@CB8	$-$117.1	77.7	$-$13.4
*trans*-V@CB8	$-$120.2	81.0	$-$13.5

All free energies of association are negative which shows that each complex may be thermodynamically favourable, but the magnitude suggests some complexes are more likely than others. The *trans*-IV@CB8 and *trans*-V@CB8 complexes have the most negative $\Delta G_a$, but it is unlikely that these stereoisomers will be thermodynamically available due to the increased relative free energy of the free cyclam (Fig. 3) and the increased strain of including these stereoisomers compared to the others studied here (Table 2). The *trans*-III@CB8 complex has the smallest $\Delta G_a$, which is in line with experimental results in that this complex is not observed. Both *trans*-I@CB8 and *trans*-II@CB8 complexes have similar $\Delta G_a$ values, and through merging these data with the free cyclam free energies and inclusion strain, we gain a deeper understanding as to why only these complexes are observed.

## Conclusions

A study of the five diastereoisomers of *trans*-Cu$^{\textrm{II}}$ cyclam and their supramolecular complexes formed with cucurbit[8]uril has been performed to interpret the unusual stabilization of high-energy cyclams. The relative free energies of the five cyclam diastereoisomers reveals that although the *trans*-III cyclam is the minimum energy conformer, both the *trans*-I and *trans*-II are comparable in energy, but *trans*-IV and *trans*-V are significantly less accessible. Since only *trans*-III is reported in the free state, the energy difference between *trans*-III and the *trans*-I and *trans*-II configurations—2.9 kcal/mol and 5.1 kcal/mol, respectively — provides insight to which diastereoisomers are thermodynamically accessible. This suggests that the cyclam distorts into other diastereoisomers when it enters the host molecule.

The crystal structures reported by Hart et. al [7] and quantum chemistry methods collectively provided insight into the interactions within the supramolecular system. The energy decomposition analysis showed that the Pauli repulsion felt by *trans*-II is much higher than *trans*-I; which may explain why *trans*-I and *trans*-II occur at a 7:3 ratio experimentally.

Visualization of the non-covalent interactions reveals that the position of the cyclam inside CB8 influences the strength of interactions. All the results above agree with the experimental findings and provide additional details explaining why *trans*-III is not included in CB8, while *trans*-I and *trans*-II are.

The computed structures showed the differences in structure and non-covalent interactions for both the experimentally observed and the other three possible complexes. The *trans*-I and *trans*-II cyclams are encapsulated inside CB8, elongating the host molecule and maximizing the non-covalent interactions. The *trans*-III, IV, and V cyclams protrude out of the cavity and do not stretch CB8 as much as the experimentally observed complexes. Even though *trans*-III is found as the only free cyclam diastereoisomers, its interaction energy and free energy of association with CB8 is smaller than if *trans*-III bends to another diastereoisomer and binds to the host molecule.

Overall, the computational methods employed in this work were able to provide a deeper understanding to this interesting chemical phenomenon. We now understand that CB8 preferentially selects *trans*-I and *trans*-II to bind due to more favourable thermochemistry and stronger intermolecular interactions relative to *trans*-III. These *trans*-I and *trans*-II complexes are more energetically favourable than complexes formed with any other diastereoisomers, due to the structural nature of inclusion. Since other diastereoisomers (*trans*-IV and *trans*-V) are inaccessible in the free state, forming these complexes is unlikely compared to the experimentally observed complexes.

### Supplementary Information

Below is the link to the electronic supplementary material.

Supplementary file 1 (pdf 117 KB)
