# Supplementary material for: Uncovering the mechanism of selective stabilization of high-energy diastereoisomers via inclusion
Source: Theor Chem Acc. 2023 Dec 12;143(1):4. doi: 10.1007/s00214-023-03077-7 (PMC10716081; doi:10.1007/s00214-023-03077-7)
Supplement: Supplementary file 1 — (pdf 117 KB) [file 214_2023_3077_MOESM1_ESM.pdf]

Supporting Information Available

Relative energies of solvent coordinated to free cyclam. QTAIM analysis of each computed complex.

Table S1: Relative enthalpies (kcal/mol) of free trans Cu^{II} cyclam computed with TPSS/def2TZVP.

	Gas phase	Solvent
t10w	1.62	1.42
t20w	4.57	3.82
t30w	0.00	0.00
t40w	11.05	10.17
t50w	7.12	6.68
t11w	1.21	2.57
t21w	7.37	8.32
t31w	0.00	0.00
t41w	11.37	11.08
t51w	6.89	7.20
t12w	4.05	1.98
t22w	7.48	6.78
t32w	0.00	0.00
t42w	11.54	10.29
t52w	8.21	6.30

Table S2: Relative Gibbs free energies (kcal/mol) of free trans Cu^{II} cyclam computed with TPSS/def2TZVP.

	Gas phase	Solvent
t10w	1.83	2.90
t20w	4.58	5.15
t30w	0.00	0.00
t40w	10.77	11.29
t50w	7.07	8.07
t11w	1.81	2.33
t21w	7.81	8.03
t31w	0.00	0.00
t41w	11.64	11.35
t51w	7.34	8.36
t12w	3.91	1.46
t22w	7.16	5.97
t32w	0.00	0.00
t42w	11.65	8.47
t52w	7.75	5.38

Table S3: Structural parameters (bond lengths (Å) and bond angles (°)) of the crystal (*trans*-I-1water and *trans*-II-2water) and computed cyclam structures within CB8 (*trans*-I through *trans*-V).

	Cu-N1	Cu-N2	Cu-N3	Cu-N4	N1-Cu-N2	N2-Cu-N3	N3-Cu-N4	N4-Cu-N1	N1-N2-N3-N4
trans-I-1water	2.050	2.041	2.050	2.041	94.6	86.3	94.6	86.3	-17.7
trans-II-2water	2.038	2.019	2.066	2.022	93.1	85.3	94.8	86.3	-18.1
trans-I	2.037	2.037	2.027	2.021	89.6	86.5	96.9	86.6	1.6
trans-II	2.025	2.028	2.027	2.029	93.6	86.2	92.7	87.5	3.5
trans-III	2.023	2.045	2.031	2.037	95.8	85.8	92.7	85.7	1.5
trans-IV	2.023	2.023	2.028	2.028	94.0	86.7	95.3	86.7	16.8
trans-V	2.021	2.016	2.043	2.035	94.0	87.1	93.2	87.2	-12.4

Table S4: Analysis of the electron density at the bond critical points for the optimized geometry at the TPSS/def2TZVP level for the *trans*-I Cu^{II}cyclam. All values are in atomic units unless specified otherwise.

Atoms	distance (Å)	ρ	$\nabla^2\rho$	V	G	K
C100 - C104	2.931927	0.245675	-0.613727	-0.261186	0.053877	0.207309
C100 - H101	2.047679	0.285328	-1.011841	-0.319583	0.033311	0.286272
C100 - N154	2.776348	0.263657	-0.634172	-0.386283	0.11387	0.272413
C100 - N155	2.713195	0.282978	-0.758807	-0.445738	0.128018	0.31772
C102 - N154	2.62834	0.312016	-0.933431	-0.555052	0.160847	0.394205
C102 - N156	2.609452	0.317267	-0.977529	-0.588926	0.172272	0.416654
C102 - O178	2.319884	0.407664	-0.447559	-1.356793	0.622452	0.734342
C103 - N155	2.632569	0.308079	-0.9147	-0.554482	0.162903	0.391578
C103 - N157	2.648774	0.303596	-0.878498	-0.528508	0.154442	0.374066
C103 - O179	2.289968	0.423121	-0.326516	-1.461832	0.690101	0.77173
C104 - H105	2.047964	0.285303	-1.011732	-0.319436	0.033252	0.286185
C104 - N156	2.731535	0.276066	-0.724	-0.432537	0.125768	0.306768
C104 - N157	2.749462	0.272766	-0.684086	-0.407062	0.11802	0.289041
C106 - H107	2.035347	0.291068	-1.067622	-0.326084	0.029589	0.296495
C106 - H108	2.049952	0.283764	-0.998537	-0.318239	0.034302	0.283936
C106 - N156	2.739515	0.265257	-0.691034	-0.440454	0.133848	0.306606
C106 - N158	2.735578	0.269478	-0.705731	-0.435679	0.129623	0.306056
C109 - H110	2.035047	0.291326	-1.07013	-0.326325	0.029396	0.296929
C109 - H111	2.05207	0.282827	-0.991705	-0.316934	0.034504	0.28243
C109 - N157	2.741387	0.268224	-0.697341	-0.428757	0.127211	0.301546
C109 - N159	2.734579	0.266876	-0.701135	-0.445019	0.134868	0.310152
C112 - C116	2.951468	0.240391	-0.587993	-0.251335	0.052168	0.199167
C112 - H113	2.046472	0.286042	-1.017266	-0.320475	0.033079	0.287396

Atoms	distance (Å)	ρ	$\nabla^2\rho$	V	G	K
C112 - N158	2.753017	0.27036	-0.676711	-0.406497	0.11866	0.287837
C112 - N159	2.727885	0.277548	-0.729573	-0.43396	0.125783	0.308177
C114 - N158	2.636045	0.307249	-0.906749	-0.54905	0.161181	0.387869
C114 - N160	2.623724	0.311749	-0.937774	-0.566977	0.166267	0.40071
C114 - O180	2.296288	0.420095	-0.361234	-1.438686	0.674189	0.764497
C115 - N159	2.618969	0.31368	-0.953764	-0.574718	0.168138	0.406579
C115 - N161	2.631578	0.310376	-0.924125	-0.551489	0.160229	0.39126
C115 - O181	2.309306	0.413856	-0.418688	-1.394381	0.644854	0.749526
C116 - H117	2.046051	0.286393	-1.019572	-0.32097	0.033038	0.287931
C116 - N160	2.731425	0.276635	-0.721916	-0.427995	0.123758	0.304237
C116 - N161	2.758241	0.269345	-0.668638	-0.401567	0.117204	0.284363
C118 - H119	2.034981	0.291242	-1.069137	-0.326287	0.029501	0.296786
C118 - H120	2.043036	0.286171	-1.017204	-0.322099	0.033899	0.2882
C118 - N160	2.744609	0.264598	-0.679884	-0.431384	0.130706	0.300677
C118 - N162	2.739919	0.266145	-0.689463	-0.435436	0.131535	0.303901
C12 - C14	2.884474	0.249178	-0.62515	-0.274289	0.059001	0.215288
C12 - H13	2.042822	0.278458	-0.953393	-0.318327	0.039989	0.278337
C12 - H39	2.045583	0.278465	-0.951897	-0.31819	0.040108	0.278082
C121 - H122	2.036452	0.290765	-1.065715	-0.325667	0.029619	0.296048
C121 - H123	2.054122	0.281704	-0.983245	-0.31549	0.03484	0.280651
C121 - N161	2.745502	0.26507	-0.68267	-0.428354	0.128843	0.299511
C121 - N163	2.731492	0.271061	-0.716548	-0.440986	0.130925	0.310062
C124 - C128	2.939315	0.243894	-0.605952	-0.257548	0.05303	0.204518
C124 - H125	2.047024	0.285777	-1.015337	-0.320042	0.033104	0.286938
C124 - N162	2.723943	0.278699	-0.737917	-0.437293	0.126407	0.310886

Atoms	distance (Å)	ρ	$\nabla^2\rho$	V	G	K
C124 - N163	2.762333	0.267931	-0.659577	-0.397272	0.116189	0.281083
C126 - N162	2.629038	0.309731	-0.926347	-0.560281	0.164347	0.395934
C126 - N164	2.639055	0.307371	-0.901174	-0.540833	0.15777	0.383063
C126 - O182	2.296521	0.419865	-0.358198	-1.438284	0.674367	0.763917
C127 - N163	2.64024	0.306627	-0.899586	-0.541327	0.158215	0.383112
C127 - N165	2.621511	0.313226	-0.949285	-0.571447	0.167063	0.404384
C127 - O183	2.298085	0.419262	-0.368056	-1.43314	0.670563	0.762577
C128 - H129	2.046297	0.286226	-1.018615	-0.320661	0.033004	0.287657
C128 - N164	2.768552	0.265825	-0.645479	-0.39073	0.11468	0.27605
C128 - N165	2.723223	0.279082	-0.739123	-0.437202	0.12621	0.310991
C130 - H131	2.049771	0.28337	-0.995846	-0.318014	0.034526	0.283488
C130 - H132	2.035815	0.290949	-1.066428	-0.326005	0.029699	0.296306
C130 - N164	2.74521	0.266574	-0.685736	-0.425196	0.126881	0.298315
C130 - N166	2.733606	0.268006	-0.705976	-0.445436	0.134471	0.310965
C133 - H134	2.035644	0.291169	-1.068958	-0.326127	0.029444	0.296683
C133 - H135	2.047054	0.284317	-1.003337	-0.319517	0.034341	0.285176
C133 - N165	2.73751	0.266891	-0.699168	-0.441129	0.133169	0.307961
C133 - N167	2.739541	0.267591	-0.693712	-0.433168	0.12987	0.303298
C14 - H15	2.04026	0.286048	-1.012116	-0.323654	0.035313	0.288342
C14 - H16	2.044569	0.283958	-0.998516	-0.319696	0.035034	0.284663
C17 - C20	2.870905	0.256597	-0.666854	-0.284473	0.05888	0.225593
C17 - H18	2.038844	0.284336	-0.997938	-0.322564	0.03654	0.286024
C17 - H19	2.040186	0.284475	-0.99722	-0.323899	0.037297	0.286602
C20 - H21	2.038231	0.286301	-1.016854	-0.323009	0.034398	0.288611
C20 - H22	2.040726	0.283946	-0.994536	-0.322906	0.037136	0.28577

Atoms	distance (Å)	ρ	$\nabla^2\rho$	V	G	K
C23 - C26	2.890026	0.247621	-0.616643	-0.271852	0.058846	0.213006
C23 - H24	2.043384	0.285036	-1.008199	-0.320358	0.034154	0.286204
C23 - H25	2.045148	0.281935	-0.980368	-0.321283	0.038095	0.283187
C26 - C29	2.890079	0.247565	-0.616134	-0.272	0.058983	0.213017
C26 - H27	2.049522	0.276987	-0.94275	-0.315442	0.039877	0.275565
C26 - H28	2.041328	0.279923	-0.966551	-0.318031	0.038197	0.279834
C29 - H30	2.045144	0.282277	-0.983429	-0.321065	0.037604	0.283461
C29 - H31	2.041146	0.284503	-0.998399	-0.322485	0.036443	0.286042
C32 - C35	2.86724	0.257211	-0.669645	-0.286341	0.059465	0.226876
C32 - H33	2.040969	0.283865	-0.994988	-0.321932	0.036592	0.285339
C32 - H34	2.038782	0.285213	-1.001323	-0.325437	0.037553	0.287884
C35 - H36	2.037382	0.287431	-1.021885	-0.325749	0.035139	0.29061
C35 - H37	2.040207	0.284826	-1.006997	-0.320763	0.034507	0.286256
C35 - N38	2.828125	0.244651	-0.546092	-0.345835	0.104656	0.241179
C40 - C44	2.95119	0.240338	-0.588165	-0.251174	0.052066	0.199108
C40 - H41	2.048367	0.285635	-1.013042	-0.320009	0.033374	0.286635
C40 - N166	2.733748	0.275436	-0.718408	-0.429167	0.124782	0.304384
C40 - N167	2.742107	0.274212	-0.69899	-0.415587	0.12042	0.295167
C42 - N136	2.630662	0.310579	-0.926715	-0.552761	0.160541	0.39222
C42 - N166	2.613164	0.315235	-0.963926	-0.584646	0.171832	0.412814
C42 - O168	2.309676	0.413358	-0.418219	-1.392252	0.643848	0.748403
C43 - N137	2.629306	0.308821	-0.919949	-0.559941	0.164977	0.394964
C43 - N167	2.63839	0.306433	-0.900385	-0.543851	0.159377	0.384473
C43 - O169	2.291593	0.422436	-0.338747	-1.455566	0.68544	0.770127
C44 - H45	2.048979	0.285157	-1.009489	-0.319434	0.033531	0.285903

Atoms	distance (Å)	ρ	$\nabla^2\rho$	V	G	K
C44 - N136	2.760528	0.268566	-0.665377	-0.399654	0.116655	0.282999
C44 - N137	2.72177	0.279976	-0.741535	-0.437765	0.126191	0.311574
C46 - H47	2.056705	0.280919	-0.976903	-0.314236	0.035005	0.279231
C46 - H48	2.034985	0.291107	-1.066932	-0.326437	0.029852	0.296585
C46 - N136	2.739248	0.268066	-0.699829	-0.432955	0.128999	0.303956
C46 - N138	2.741341	0.268016	-0.697594	-0.429291	0.127446	0.301845
C49 - H50	2.047882	0.284379	-1.003259	-0.319518	0.034352	0.285167
C49 - H51	2.035737	0.290974	-1.069405	-0.325675	0.029162	0.296513
C49 - N137	2.734788	0.267953	-0.701145	-0.441856	0.133285	0.308571
C49 - N139	2.737397	0.266636	-0.695344	-0.440633	0.133398	0.307235
C52 - C56	2.936038	0.244452	-0.607762	-0.258929	0.053494	0.205435
C52 - H53	2.048306	0.285125	-1.009878	-0.319377	0.033454	0.285923
C52 - N138	2.767879	0.266674	-0.650172	-0.392184	0.11482	0.277364
C52 - N139	2.716117	0.281637	-0.753877	-0.445203	0.128367	0.316836
C54 - N138	2.637742	0.308425	-0.909442	-0.541737	0.157188	0.384549
C54 - N140	2.618102	0.313577	-0.952788	-0.576744	0.169274	0.407471
C54 - O170	2.306088	0.414988	-0.399003	-1.404679	0.652464	0.752215
C55 - N139	2.627024	0.309941	-0.927316	-0.563256	0.165714	0.397543
C55 - N141	2.65049	0.303723	-0.87645	-0.523828	0.152358	0.371471
C55 - O171	2.294181	0.421098	-0.347196	-1.446991	0.680096	0.766895
C56 - H57	2.048321	0.285137	-1.010009	-0.319365	0.033431	0.285934
C56 - N140	2.724159	0.278601	-0.738456	-0.439152	0.127269	0.311883
C56 - N141	2.758774	0.270002	-0.667138	-0.398641	0.115928	0.282713
C58 - H59	2.035225	0.291172	-1.068789	-0.326255	0.029529	0.296726
C58 - H60	2.051299	0.283001	-0.99284	-0.31737	0.03458	0.28279

Atoms	distance (Å)	ρ	$\nabla^2\rho$	V	G	K
C58 - N140	2.728357	0.269384	-0.716466	-0.452865	0.136874	0.315991
C58 - N142	2.74591	0.266636	-0.685722	-0.423926	0.126247	0.297678
C61 - H62	2.035295	0.291181	-1.069619	-0.326166	0.029381	0.296786
C61 - H63	2.052199	0.282659	-0.990195	-0.316853	0.034652	0.282201
C61 - N141	2.750454	0.265462	-0.677655	-0.418005	0.124295	0.293709
C61 - N143	2.730682	0.268615	-0.711335	-0.449696	0.135931	0.313765
C64 - C68	2.9465	0.241678	-0.594658	-0.253617	0.052476	0.201141
C64 - H65	2.048335	0.285335	-1.011246	-0.319564	0.033376	0.286188
C64 - N142	2.756423	0.27018	-0.671472	-0.401493	0.116813	0.284681
C64 - N143	2.727362	0.277756	-0.731746	-0.434422	0.125743	0.308679
C66 - N142	2.641728	0.306382	-0.896623	-0.537509	0.156677	0.380832
C66 - N144	2.626336	0.310256	-0.929375	-0.564519	0.166088	0.398431
C66 - O172	2.294548	0.420835	-0.348689	-1.445241	0.679035	0.766207
C67 - N143	2.618327	0.313413	-0.951627	-0.576462	0.169278	0.407184
C67 - N145	2.635164	0.308682	-0.912874	-0.546852	0.159317	0.387535
C67 - O173	2.304291	0.416226	-0.397277	-1.411328	0.656004	0.755324
C68 - H69	2.047768	0.285735	-1.014079	-0.320121	0.033301	0.28682
C68 - N144	2.72375	0.278835	-0.737563	-0.437351	0.12648	0.310871
C68 - N145	2.759351	0.268982	-0.666491	-0.400069	0.116723	0.283346
C70 - H71	2.043272	0.285946	-1.015707	-0.321841	0.033957	0.287884
C70 - H72	2.035069	0.291233	-1.06877	-0.326279	0.029543	0.296736
C70 - N144	2.737315	0.267136	-0.695337	-0.438986	0.132576	0.30641
C70 - N146	2.740846	0.265808	-0.68818	-0.435733	0.131844	0.303889
C73 - H74	2.036893	0.290533	-1.064632	-0.325273	0.029557	0.295715
C73 - H75	2.053667	0.281734	-0.983306	-0.315623	0.034898	0.280725

Atoms	distance (Å)	ρ	$\nabla^2\rho$	V	G	K
C73 - N145	2.744051	0.266326	-0.688651	-0.428681	0.128259	0.300422
C73 - N147	2.739776	0.268087	-0.698425	-0.432295	0.128844	0.303451
C76 - C80	2.944473	0.242418	-0.598835	-0.254809	0.05255	0.202259
C76 - H77	2.046797	0.286065	-1.017146	-0.320462	0.033088	0.287374
C76 - N146	2.728968	0.276995	-0.726824	-0.431846	0.12507	0.306776
C76 - N147	2.760508	0.268678	-0.66311	-0.398049	0.116136	0.281913
C78 - N146	2.622057	0.312461	-0.943439	-0.570383	0.167261	0.403121
C78 - N148	2.637784	0.307362	-0.904806	-0.544713	0.159256	0.385457
C78 - O174	2.297442	0.419494	-0.365067	-1.434961	0.671847	0.763114
C79 - N147	2.642019	0.306032	-0.894213	-0.537448	0.156948	0.380501
C79 - N149	2.625678	0.311106	-0.936139	-0.565403	0.165684	0.399719
C79 - O175	2.296547	0.419961	-0.359009	-1.43854	0.674394	0.764146
C80 - H81	2.046942	0.285996	-1.016683	-0.320389	0.033109	0.28728
C80 - N148	2.761935	0.267469	-0.658601	-0.398285	0.116817	0.281467
C80 - N149	2.722515	0.279428	-0.741123	-0.437955	0.126337	0.311618
C82 - H83	2.035566	0.291123	-1.066814	-0.3263	0.029798	0.296502
C82 - H84	2.04838	0.283989	-1.000819	-0.318788	0.034292	0.284497
C82 - N148	2.731626	0.271157	-0.713131	-0.438756	0.130236	0.308519
C82 - N150	2.739072	0.26566	-0.694166	-0.442945	0.134702	0.308244
C85 - H86	2.048426	0.283822	-0.999371	-0.318774	0.034466	0.284309
C85 - H87	2.035815	0.291118	-1.069396	-0.325981	0.029316	0.296665
C85 - N149	2.734879	0.267717	-0.703957	-0.442916	0.133464	0.309453
C85 - N151	2.750767	0.264269	-0.672283	-0.420425	0.126177	0.294248
C88 - C92	2.94377	0.242435	-0.598064	-0.255072	0.052778	0.202294
C88 - H89	2.046899	0.286071	-1.017219	-0.320434	0.033065	0.287369

Atoms	distance (Å)	ρ	$\nabla^2\rho$	V	G	K
C88 - N150	2.732499	0.275306	-0.721143	-0.432613	0.126164	0.306449
C88 - N151	2.748404	0.272972	-0.685753	-0.407214	0.117888	0.289326
C9 - C12	2.885223	0.24905	-0.624599	-0.274299	0.059075	0.215224
C9 - H10	2.046997	0.281949	-0.981723	-0.318796	0.036682	0.282113
C9 - H11	2.041206	0.285304	-1.005609	-0.32286	0.035729	0.287131
C9 - N38	2.823373	0.244622	-0.554598	-0.353186	0.107268	0.245918
C90 - N150	2.597631	0.32183	-1.008347	-0.610138	0.179025	0.431112
C90 - N152	2.626758	0.313136	-0.941983	-0.557103	0.160803	0.396299
C90 - O176	2.322481	0.407142	-0.470021	-1.348983	0.615739	0.733244
C91 - N151	2.64628	0.30425	-0.882531	-0.531056	0.155212	0.375845
C91 - N153	2.63598	0.306543	-0.90519	-0.550159	0.16193	0.388228
C91 - O177	2.288031	0.424041	-0.316355	-1.468737	0.694824	0.773913
C92 - H93	2.047739	0.285428	-1.012193	-0.319772	0.033362	0.28641
C92 - N152	2.779737	0.262579	-0.627858	-0.382809	0.112922	0.269887
C92 - N153	2.711079	0.283679	-0.763946	-0.448206	0.12861	0.319596
C94 - H95	2.033981	0.291326	-1.066019	-0.327118	0.030307	0.296812
C94 - H96	2.057156	0.280959	-0.97719	-0.31402	0.034861	0.279159
C94 - N152	2.743092	0.267422	-0.692718	-0.425944	0.126382	0.299562
C94 - N154	2.737284	0.269114	-0.706276	-0.434588	0.129009	0.305578
C97 - H98	2.035716	0.290967	-1.070045	-0.325563	0.029026	0.296537
C97 - H99	2.046743	0.284902	-1.007133	-0.320273	0.034245	0.286028
C97 - N153	2.734393	0.267996	-0.702382	-0.442701	0.133553	0.309148
C97 - N155	2.741055	0.265184	-0.685907	-0.436537	0.13253	0.304007
Cu1 - N3	3.831759	0.087654	0.291949	-0.126257	0.099622	0.026635
Cu1 - N38	3.820906	0.088931	0.294329	-0.128515	0.101048	0.027466

Atoms	distance (Å)	ρ	$\nabla^2\rho$	V	G	K
Cu1 - N5	3.84992	0.084979	0.292739	-0.122826	0.098006	0.024821
Cu1 - N7	3.849124	0.084947	0.292409	-0.122613	0.097858	0.024755
H15 - O170	4.515846	0.009839	0.037573	-0.005444	0.007418	-0.001975
H16 - O172	6.058556	0.002178	0.008535	-0.000944	0.001539	-0.000595
H16 - O174	5.627841	0.002879	0.010687	-0.001338	0.002005	-0.000667
H18 - C78	5.130912	0.005692	0.019145	-0.003132	0.003959	-0.000827
H18 - N144	6.281514	0.002819	0.011311	-0.001289	0.002058	-0.00077
H18 - N147	6.086068	0.003097	0.010758	-0.001321	0.002005	-0.000684
H19 - N142	5.900473	0.003461	0.012161	-0.00152	0.00228	-0.00076
H2 - N38	1.915423	0.32749	-1.629611	-0.507192	0.049895	0.457297
H2 - O178	3.774481	0.022241	0.084453	-0.01618	0.018647	-0.002467
H21 - O173	4.727085	0.009036	0.03356	-0.004868	0.006629	-0.001761
H21 - O175	5.782829	0.004892	0.020755	-0.002648	0.003918	-0.001271
H24 - O171	6.716658	0.001541	0.006485	-0.00067	0.001145	-0.000476
H24 - O173	4.934524	0.006812	0.0252	-0.003539	0.004919	-0.001381
H25 - N138	7.042236	0.001052	0.003834	-0.000367	0.000663	-0.000296
H25 - N141	6.504051	0.001886	0.006933	-0.000736	0.001235	-0.000499
H28 - O169	6.122717	0.002021	0.00771	-0.0009	0.001414	-0.000514
H28 - O171	6.081498	0.001981	0.00762	-0.000853	0.001379	-0.000526
H30 - O168	5.995287	0.00366	0.013319	-0.001846	0.002588	-0.000742
H31 - O183	5.221506	0.007587	0.026511	-0.004056	0.005342	-0.001286
H33 - N161	5.608252	0.005908	0.020837	-0.003015	0.004112	-0.001097
H33 - N162	6.40017	0.005805	0.020501	-0.003118	0.004122	-0.001004
H33 - N163	5.481738	0.005049	0.015491	-0.002354	0.003113	-0.00076
H34 - N158	5.111873	0.007913	0.026114	-0.004045	0.005287	-0.001242

Atoms	distance (Å)	ρ	$\nabla^2\rho$	V	G	K
H36 - O168	4.315549	0.012682	0.048246	-0.007388	0.009725	-0.002337
H37 - O180	5.441046	0.003915	0.015305	-0.001899	0.002862	-0.000964
H37 - O182	5.535706	0.004795	0.020223	-0.002562	0.003809	-0.001247
H39 - O176	5.072252	0.005967	0.024564	-0.003354	0.004748	-0.001394
H4 - O176	3.706813	0.025318	0.089931	-0.019184	0.020834	-0.001649
H8 - O181	4.553434	0.00943	0.035472	-0.004782	0.006825	-0.002043
N3 - C14	2.828451	0.241906	-0.540883	-0.352629	0.108704	0.243925
N3 - C17	2.817524	0.249503	-0.568065	-0.349599	0.103791	0.245807
N3 - H4	1.917201	0.326577	-1.614518	-0.505193	0.050782	0.454412
N5 - C20	2.823874	0.245715	-0.555055	-0.350211	0.105724	0.244488
N5 - C23	2.838199	0.240882	-0.531133	-0.340332	0.103774	0.236558
N5 - H6	1.897384	0.336503	-1.630872	-0.513332	0.052807	0.460525
N7 - C29	2.830687	0.243857	-0.545074	-0.344452	0.104092	0.24036
N7 - C32	2.819342	0.246999	-0.561485	-0.353817	0.106723	0.247094
N7 - H8	1.903232	0.333645	-1.642181	-0.512831	0.051143	0.461688
O172 - O174	6.28591	0.004452	0.017603	-0.002141	0.003271	-0.00113

Table S5: Analysis of the electron density at the bond critical points for the optimized geometry at the TPSS/def2TZVP level for the *trans*-II Cu^{II}cyclam. All values are in atomic units unless specified otherwise.

Atoms	distance (Å)	ρ	$\nabla^2\rho$	V	G	K
O175 - O177	6.226183	0.004518	0.018093	-0.002184	0.003354	-0.001169
N9 - H10	1.900818	0.334787	-1.631191	-0.512529	0.052365	0.460163
N9 - C34	2.827822	0.245821	-0.551352	-0.343357	0.102759	0.240597

Atoms	distance (Å)	ρ	$\nabla^2\rho$	V	G	K
N9 - C31	2.833845	0.241053	-0.536545	-0.346556	0.10621	0.240346
N7 - H8	1.908258	0.330759	-1.655802	-0.513524	0.049787	0.463737
N7 - C25	2.834112	0.241936	-0.538281	-0.343667	0.104548	0.239118
N7 - C22	2.828249	0.245457	-0.5508	-0.343351	0.102825	0.240525
N5 - H6	1.91181	0.328726	-1.624048	-0.507387	0.050687	0.456699
N5 - C19	2.814071	0.249534	-0.572822	-0.355217	0.106006	0.249211
N5 - C16	2.822616	0.244467	-0.55588	-0.355597	0.108314	0.247284
N2 - H4	1.912785	0.328657	-1.622505	-0.506128	0.050251	0.455877
N2 - C37	2.816721	0.248537	-0.567185	-0.354276	0.10624	0.248036
N2 - C11	2.813945	0.247066	-0.5703	-0.362852	0.110138	0.252713
H8 - O179	4.021155	0.016468	0.063154	-0.010205	0.012997	-0.002792
H6 - O176	3.938329	0.019233	0.070307	-0.012559	0.015068	-0.002509
H4 - O168	3.936553	0.018705	0.071107	-0.012427	0.015102	-0.002675
H39 - O180	7.614432	0.001695	0.007479	-0.000798	0.001334	-0.000536
H39 - O178	6.289062	0.001685	0.006283	-0.000707	0.001139	-0.000432
H38 - O182	4.313985	0.013217	0.051375	-0.008033	0.010438	-0.002406
H36 - N166	5.496811	0.004692	0.015928	-0.002257	0.003119	-0.000862
H36 - N164	5.945151	0.004019	0.015314	-0.001911	0.00287	-0.000959
H35 - N163	5.501767	0.00498	0.015819	-0.002284	0.003119	-0.000836
H35 - N160	5.783257	0.003727	0.011959	-0.001649	0.00232	-0.00067
H35 - C126	6.08844	0.003689	0.015292	-0.002063	0.002943	-0.00088
H35 - C115	5.21639	0.005841	0.02074	-0.003325	0.004255	-0.00093
H33 - O183	5.380088	0.003808	0.013716	-0.001778	0.002603	-0.000825
H33 - O181	5.282308	0.006334	0.026987	-0.003746	0.005246	-0.0015
H32 - O171	6.351173	0.001793	0.007242	-0.000783	0.001297	-0.000514

Atoms	distance (Å)	ρ	$\nabla^2\rho$	V	G	K
H32 - N137	6.639869	0.001451	0.005075	-0.000533	0.000901	-0.000368
H3 - C14	2.040111	0.281056	-0.970513	-0.321022	0.039197	0.281825
H27 - O171	5.260298	0.004967	0.018947	-0.002546	0.003642	-0.001095
H24 - N153	5.86642	0.003723	0.012832	-0.001735	0.002471	-0.000737
H24 - N150	6.151348	0.002624	0.009022	-0.001066	0.001661	-0.000595
H23 - O175	5.554811	0.003622	0.014213	-0.001721	0.002637	-0.000916
H23 - O173	5.071037	0.006216	0.024233	-0.003323	0.004691	-0.001368
H21 - O173	5.80302	0.004709	0.018721	-0.002568	0.003624	-0.001056
H20 - N147	5.834462	0.003615	0.011754	-0.00155	0.002244	-0.000694
H20 - C78	5.256821	0.006328	0.021602	-0.003465	0.004433	-0.000968
H18 - O174	4.93233	0.00645	0.02472	-0.003436	0.004808	-0.001372
H18 - O172	6.385005	0.001408	0.005407	-0.000573	0.000962	-0.00039
H17 - O170	5.765676	0.003024	0.011924	-0.001424	0.002203	-0.000778
H12 - O178	6.4061	0.001397	0.005043	-0.000551	0.000906	-0.000355
H12 - O176	5.122984	0.005645	0.021951	-0.003038	0.004263	-0.001225
H10 - O181	4.710801	0.009662	0.038971	-0.00549	0.007616	-0.002127
Cu1 - N9	3.830715	0.087363	0.297847	-0.12725	0.100856	0.026394
Cu1 - N7	3.83448	0.087206	0.293626	-0.125881	0.099644	0.026237
Cu1 - N5	3.827437	0.087803	0.297382	-0.12747	0.100908	0.026562
Cu1 - N2	3.832027	0.087391	0.295105	-0.126479	0.100128	0.026351
C97 - N155	2.733698	0.266676	-0.702189	-0.448552	0.136503	0.31205
C97 - N153	2.737745	0.269803	-0.706335	-0.431596	0.127506	0.30409
C97 - H99	2.052358	0.282926	-0.992222	-0.316917	0.034431	0.282486
C97 - H98	2.034914	0.2913	-1.069942	-0.326324	0.029419	0.296905
C94 - N154	2.744466	0.267541	-0.692438	-0.424049	0.12547	0.298579

Atoms	distance (Å)	ρ	$\nabla^2\rho$	V	G	K
C94 - N152	2.734203	0.266989	-0.70244	-0.445591	0.134991	0.310601
C94 - H96	2.052694	0.282831	-0.991463	-0.316701	0.034417	0.282283
C94 - H95	2.034367	0.291408	-1.069934	-0.326587	0.029552	0.297035
C92 - N153	2.750019	0.272276	-0.683114	-0.407154	0.118188	0.288966
C92 - N152	2.733064	0.275602	-0.720258	-0.43032	0.125128	0.305192
C92 - H93	2.04765	0.285396	-1.012403	-0.319567	0.033233	0.286334
C91 - O177	2.290617	0.422777	-0.32951	-1.45929	0.688456	0.770834
C91 - N153	2.64787	0.303739	-0.881349	-0.531195	0.155429	0.375766
C91 - N151	2.626997	0.310454	-0.928774	-0.562265	0.165036	0.397229
C90 - O176	2.318669	0.409252	-0.458691	-1.362431	0.623879	0.738552
C90 - N152	2.610774	0.316733	-0.974922	-0.587627	0.171948	0.415679
C90 - N150	2.624633	0.31352	-0.943614	-0.560483	0.16229	0.398193
C88 - N151	2.721354	0.280207	-0.742717	-0.437363	0.125842	0.311521
C88 - N150	2.767382	0.266505	-0.65146	-0.393922	0.115529	0.278394
C88 - H89	2.046506	0.286185	-1.017977	-0.320677	0.033092	0.287586
C88 - C92	2.943262	0.242542	-0.598221	-0.255379	0.052912	0.202467
C85 - N151	2.746511	0.263772	-0.67533	-0.430294	0.130731	0.299563
C85 - N149	2.736693	0.267402	-0.69685	-0.438225	0.132006	0.306219
C85 - H87	2.035031	0.291286	-1.069624	-0.326272	0.029433	0.296839
C85 - H86	2.042613	0.286234	-1.017816	-0.322287	0.033917	0.288371
C82 - N150	2.752556	0.26305	-0.669466	-0.421048	0.126841	0.294207
C82 - N148	2.723131	0.273835	-0.733563	-0.449496	0.133052	0.316443
C82 - H84	2.054009	0.281771	-0.983667	-0.315557	0.03482	0.280737
C82 - H83	2.036236	0.290734	-1.064072	-0.325835	0.029909	0.295927
C80 - N149	2.721012	0.279758	-0.74308	-0.439259	0.126744	0.312514

Atoms	distance (Å)	ρ	$\nabla^2\rho$	V	G	K
C80 - N148	2.766393	0.266059	-0.650445	-0.394668	0.116028	0.27864
C80 - H81	2.046352	0.286042	-1.017533	-0.320448	0.033032	0.287416
C79 - O175	2.293107	0.421585	-0.342306	-1.450483	0.682453	0.76803
C79 - N149	2.63207	0.308751	-0.920401	-0.556106	0.163003	0.393103
C79 - N147	2.639441	0.307009	-0.899492	-0.541037	0.158082	0.382955
C78 - O174	2.301623	0.417352	-0.3823	-1.420209	0.662317	0.757892
C78 - N148	2.636671	0.307705	-0.908009	-0.547486	0.160242	0.387244
C78 - N146	2.618022	0.314965	-0.960467	-0.575787	0.167835	0.407952
C76 - N147	2.76501	0.266899	-0.651659	-0.393519	0.115302	0.278217
C76 - N146	2.727366	0.277775	-0.731441	-0.433128	0.125134	0.307994
C76 - H77	2.04556	0.286568	-1.021286	-0.321079	0.032879	0.2882
C76 - C80	2.940024	0.243745	-0.605403	-0.25723	0.052939	0.20429
C73 - N147	2.745193	0.266217	-0.683623	-0.425877	0.127486	0.298391
C73 - N145	2.73368	0.268132	-0.706489	-0.445214	0.134296	0.310918
C73 - H75	2.048312	0.283773	-0.99908	-0.318748	0.034489	0.284259
C73 - H74	2.036081	0.290959	-1.066772	-0.325963	0.029635	0.296328
C70 - N146	2.741033	0.265932	-0.69319	-0.437151	0.131927	0.305224
C70 - N144	2.735373	0.268976	-0.701731	-0.437241	0.130904	0.306337
C70 - H72	2.035625	0.291181	-1.067834	-0.32625	0.029646	0.296604
C70 - H71	2.045985	0.284645	-1.005938	-0.320029	0.034272	0.285757
C68 - N145	2.729331	0.276847	-0.727305	-0.433347	0.125761	0.307587
C68 - N144	2.746584	0.272417	-0.689088	-0.411615	0.119671	0.291943
C68 - H69	2.047874	0.2859	-1.015012	-0.32035	0.033299	0.287052
C67 - O173	2.304695	0.415836	-0.395248	-1.409884	0.655536	0.754348
C67 - N145	2.61559	0.314333	-0.957878	-0.581404	0.170967	0.410437

Atoms	distance (Å)	ρ	$\nabla^2\rho$	V	G	K
C67 - N143	2.639358	0.307737	-0.905715	-0.539362	0.156467	0.382896
C66 - O172	2.292174	0.422052	-0.339305	-1.453365	0.68427	0.769096
C66 - N144	2.635255	0.307538	-0.908562	-0.549022	0.160941	0.388081
C66 - N142	2.627309	0.309656	-0.926488	-0.5636	0.165989	0.397611
C64 - N143	2.764728	0.267553	-0.656876	-0.394512	0.115147	0.279366
C64 - N142	2.722298	0.279418	-0.739959	-0.43793	0.12647	0.31146
C64 - H65	2.048583	0.285341	-1.010824	-0.319697	0.033496	0.286201
C64 - C68	2.951549	0.240289	-0.588194	-0.251011	0.051981	0.19903
C61 - N143	2.743091	0.267263	-0.69268	-0.426975	0.126903	0.300073
C61 - N141	2.742577	0.267477	-0.695307	-0.428677	0.127425	0.301252
C61 - H63	2.056759	0.280842	-0.976228	-0.314155	0.035049	0.279106
C61 - H62	2.035413	0.290985	-1.066477	-0.326155	0.029768	0.296387
C58 - N142	2.728648	0.270116	-0.714345	-0.448541	0.134977	0.313564
C58 - N140	2.7391	0.265808	-0.690788	-0.439804	0.133554	0.306251
C58 - H60	2.046516	0.284902	-1.007243	-0.320318	0.034254	0.286065
C58 - H59	2.035524	0.291036	-1.068776	-0.325856	0.029331	0.296525
C56 - N141	2.768147	0.26679	-0.64904	-0.391298	0.114519	0.276779
C56 - N140	2.717228	0.281121	-0.751486	-0.444642	0.128385	0.316257
C56 - H57	2.047935	0.285189	-1.010714	-0.319437	0.033379	0.286057
C55 - O171	2.301852	0.417142	-0.38007	-1.419908	0.662445	0.757463
C55 - N141	2.642798	0.306509	-0.894761	-0.534395	0.155353	0.379043
C55 - N139	2.62599	0.310645	-0.933764	-0.56447	0.165514	0.398955
C54 - O170	2.296969	0.419623	-0.358193	-1.437124	0.673788	0.763336
C54 - N140	2.622348	0.311919	-0.940516	-0.570156	0.167514	0.402642
C54 - N138	2.652078	0.303533	-0.875329	-0.522428	0.151798	0.37063

Atoms	distance (Å)	ρ	$\nabla^2\rho$	V	G	K
C52 - N139	2.718394	0.28081	-0.750521	-0.444057	0.128213	0.315844
C52 - N138	2.767081	0.267304	-0.650512	-0.391579	0.114476	0.277104
C52 - H53	2.048617	0.284703	-1.007027	-0.318829	0.033536	0.285293
C52 - C56	2.927292	0.24692	-0.61975	-0.263584	0.054323	0.209261
C49 - N139	2.726528	0.269824	-0.719997	-0.45406	0.137031	0.31703
C49 - N137	2.748022	0.265953	-0.684011	-0.422232	0.125615	0.296617
C49 - H51	2.034667	0.291333	-1.071109	-0.326317	0.02927	0.297047
C49 - H50	2.052964	0.282636	-0.989781	-0.316651	0.034603	0.282048
C46 - N138	2.742098	0.269166	-0.69992	-0.424275	0.124647	0.299627
C46 - N136	2.733951	0.26714	-0.703813	-0.446616	0.135332	0.311285
C46 - H48	2.034147	0.291429	-1.069543	-0.326751	0.029683	0.297068
C46 - H47	2.055163	0.281869	-0.984013	-0.315354	0.034676	0.280679
C44 - N137	2.745546	0.274079	-0.693143	-0.410477	0.118596	0.291882
C44 - N136	2.735025	0.275283	-0.717233	-0.428581	0.124636	0.303945
C44 - H45	2.048716	0.285042	-1.009003	-0.319206	0.033477	0.285728
C43 - O169	2.290847	0.422672	-0.331213	-1.45839	0.687793	0.770597
C43 - N167	2.632127	0.308103	-0.914548	-0.555462	0.163413	0.392049
C43 - N137	2.642655	0.305653	-0.893524	-0.537398	0.157008	0.380389
C42 - O168	2.318933	0.408652	-0.450199	-1.36117	0.62431	0.73686
C42 - N166	2.629892	0.311543	-0.930996	-0.552136	0.159694	0.392443
C42 - N136	2.609847	0.317014	-0.975467	-0.58842	0.172277	0.416143
C40 - N167	2.71752	0.281353	-0.750316	-0.4422	0.12731	0.314889
C40 - N166	2.766676	0.26669	-0.653245	-0.394808	0.115748	0.279059
C40 - H41	2.047603	0.285803	-1.014316	-0.320332	0.033377	0.286956
C40 - C44	2.944493	0.242012	-0.595495	-0.254502	0.052814	0.201688

Atoms	distance (Å)	ρ	$\nabla^2\rho$	V	G	K
C37 - H39	2.044125	0.282311	-0.984367	-0.320574	0.037241	0.283333
C37 - H38	2.039475	0.286465	-1.022752	-0.322215	0.033263	0.288951
C34 - H36	2.039946	0.284597	-0.997739	-0.323897	0.037231	0.286666
C34 - H35	2.038367	0.284518	-0.9995	-0.323221	0.036673	0.286548
C34 - C37	2.877552	0.254682	-0.656205	-0.281129	0.058539	0.22259
C31 - H33	2.042444	0.285352	-1.010254	-0.32089	0.034163	0.286727
C31 - H32	2.044847	0.282314	-0.984278	-0.320855	0.037393	0.283462
C28 - H30	2.040864	0.279865	-0.965508	-0.318617	0.03862	0.279997
C28 - H29	2.047745	0.277726	-0.947016	-0.316615	0.039931	0.276685
C28 - C31	2.887267	0.248293	-0.619959	-0.273332	0.059171	0.214161
C25 - O173	6.808819	0.003108	0.013107	-0.00151	0.002393	-0.000883
C25 - H27	2.041194	0.284136	-0.996061	-0.322773	0.036879	0.285894
C25 - H26	2.043955	0.283695	-0.995559	-0.320075	0.035593	0.284482
C25 - C28	2.890278	0.247547	-0.616528	-0.271597	0.058733	0.212865
C22 - H24	2.040451	0.283726	-0.991464	-0.323287	0.037711	0.285577
C22 - H23	2.039973	0.285697	-1.015476	-0.321074	0.033603	0.287471
C19 - H21	2.041142	0.284	-0.994474	-0.323045	0.037213	0.285832
C19 - H20	2.039288	0.284231	-0.997684	-0.322564	0.036571	0.285992
C19 - C22	2.876485	0.255206	-0.659549	-0.281736	0.058424	0.223312
C16 - H18	2.04219	0.285168	-1.007956	-0.320986	0.034499	0.286488
C16 - H17	2.04264	0.283535	-0.989949	-0.323333	0.037923	0.28541
C14 - H15	2.041715	0.280283	-0.964923	-0.320089	0.039429	0.28066
C14 - C16	2.903859	0.243587	-0.597798	-0.26436	0.057455	0.206904
C133 - N167	2.736578	0.267417	-0.697359	-0.440075	0.132868	0.307207
C133 - N165	2.74178	0.265351	-0.685768	-0.434539	0.131548	0.302991

Atoms	distance (Å)	ρ	$\nabla^2\rho$	V	G	K
C133 - H135	2.043614	0.285806	-1.014562	-0.321684	0.034022	0.287662
C133 - H134	2.035332	0.291202	-1.069175	-0.326117	0.029411	0.296705
C130 - N166	2.753782	0.263022	-0.667665	-0.417757	0.12542	0.292337
C130 - N164	2.726796	0.272009	-0.72462	-0.447533	0.133189	0.314344
C130 - H132	2.036107	0.290761	-1.064511	-0.325819	0.029846	0.295974
C130 - H131	2.053074	0.282215	-0.986758	-0.316177	0.034744	0.281433
C128 - N165	2.721704	0.279466	-0.74125	-0.438386	0.126537	0.311849
C128 - N164	2.764629	0.26633	-0.653597	-0.396142	0.116371	0.279771
C128 - H129	2.046171	0.286364	-1.019662	-0.320812	0.032948	0.287864
C127 - O183	2.293151	0.421557	-0.342031	-1.450388	0.68244	0.767948
C127 - N165	2.623361	0.31183	-0.940992	-0.569521	0.167137	0.402385
C127 - N163	2.645354	0.305005	-0.887197	-0.532825	0.155513	0.377312
C126 - O182	2.302082	0.417144	-0.38621	-1.418313	0.66088	0.757433
C126 - N164	2.625675	0.311118	-0.931928	-0.564714	0.165866	0.398848
C126 - N162	2.62263	0.313397	-0.950834	-0.56896	0.165626	0.403335
C124 - N163	2.763086	0.267312	-0.655559	-0.395682	0.115896	0.279786
C124 - N162	2.730009	0.277056	-0.726953	-0.42996	0.124111	0.305849
C124 - H125	2.045832	0.28659	-1.021053	-0.321112	0.032924	0.288188
C124 - C128	2.945702	0.242243	-0.598173	-0.254363	0.05241	0.201953
C121 - N163	2.738763	0.268756	-0.697083	-0.430897	0.128313	0.302584
C121 - N161	2.740315	0.265777	-0.693587	-0.440461	0.133532	0.306929
C121 - H123	2.046505	0.284411	-1.00413	-0.319629	0.034298	0.285331
C121 - H122	2.035947	0.291081	-1.06728	-0.326149	0.029665	0.296485
C118 - N162	2.736295	0.267943	-0.704328	-0.440315	0.132117	0.308199
C118 - N160	2.751878	0.263813	-0.668753	-0.419508	0.12616	0.293348

Atoms	distance (Å)	ρ	$\nabla^2\rho$	V	G	K
C118 - H120	2.045431	0.284786	-1.006938	-0.320263	0.034264	0.285999
C118 - H119	2.035458	0.291266	-1.069054	-0.326357	0.029547	0.29681
C116 - N161	2.732985	0.275631	-0.720549	-0.429774	0.124818	0.304956
C116 - N160	2.752556	0.271147	-0.677075	-0.404225	0.117478	0.286747
C116 - H117	2.046486	0.286487	-1.019688	-0.321012	0.033045	0.287967
C115 - O181	2.308651	0.414174	-0.416859	-1.396681	0.646233	0.750448
C115 - N161	2.60718	0.318194	-0.981763	-0.593517	0.174038	0.419479
C115 - N159	2.638215	0.308399	-0.911093	-0.542322	0.157274	0.385047
C114 - O180	2.290087	0.423038	-0.326768	-1.4613	0.689804	0.771496
C114 - N160	2.644313	0.305131	-0.887899	-0.533226	0.155625	0.3776
C114 - N158	2.63186	0.307834	-0.913821	-0.55667	0.164107	0.392562
C112 - N159	2.768159	0.266238	-0.650153	-0.392605	0.115033	0.277572
C112 - N158	2.717689	0.281201	-0.750528	-0.442441	0.127404	0.315036
C112 - H113	2.048239	0.285291	-1.010965	-0.319549	0.033404	0.286145
C112 - C116	2.947687	0.241345	-0.593035	-0.252987	0.052364	0.200623
C11 - H13	2.044615	0.28288	-0.989001	-0.319558	0.036154	0.283404
C11 - H12	2.040665	0.285422	-1.004933	-0.323938	0.036352	0.287586
C11 - C14	2.90401	0.24368	-0.598275	-0.264369	0.0574	0.206969
C109 - N159	2.737818	0.269428	-0.704775	-0.431783	0.127795	0.303988
C109 - N157	2.742112	0.267201	-0.694634	-0.429967	0.128154	0.301813
C109 - H111	2.056873	0.280972	-0.977293	-0.31404	0.034858	0.279182
C109 - H110	2.035193	0.291016	-1.065354	-0.326366	0.030013	0.296352
C106 - N158	2.733713	0.268373	-0.703773	-0.44252	0.133288	0.309231
C106 - N156	2.742371	0.264649	-0.683538	-0.435737	0.132426	0.303311
C106 - H108	2.047031	0.284877	-1.006917	-0.32011	0.034191	0.28592

Atoms	distance (Å)	ρ	$\nabla^2\rho$	V	G	K
C106 - H107	2.035573	0.290992	-1.069866	-0.325662	0.029098	0.296564
C104 - N157	2.771385	0.265716	-0.644327	-0.390065	0.114491	0.275573
C104 - N156	2.71688	0.281635	-0.752043	-0.443229	0.127609	0.31562
C104 - H105	2.046833	0.285569	-1.013862	-0.319933	0.033234	0.286699
C103 - O179	2.315784	0.410278	-0.438792	-1.372031	0.631167	0.740865
C103 - N157	2.635432	0.30972	-0.917804	-0.54455	0.157549	0.387
C103 - N155	2.61244	0.316163	-0.970102	-0.584339	0.170907	0.413432
C102 - O178	2.29456	0.420907	-0.350083	-1.445378	0.678929	0.766449
C102 - N156	2.628249	0.309702	-0.924852	-0.560505	0.164646	0.395859
C102 - N154	2.649147	0.303779	-0.878861	-0.527409	0.153847	0.373562
C100 - N155	2.72626	0.277996	-0.734344	-0.437334	0.126874	0.31046
C100 - N154	2.756222	0.270649	-0.671351	-0.401472	0.116817	0.284655
C100 - H101	2.047149	0.285416	-1.012848	-0.319656	0.033222	0.286434
C100 - C104	2.930677	0.245898	-0.614268	-0.261799	0.054116	0.207683

Table S6: Analysis of the electron density at the bond critical points for the optimized geometry at TPSS/def2TZVP for the *trans*-III Cu^{II}cyclam. All values are in atomic units unless specified otherwise.

Atoms	distance (Å)	ρ	$\nabla^2\rho$	V	G	K
C1 - C5	2.949588	0.241045	-0.592153	-0.252255	0.052108	0.200147
C1 - H2	2.047126	0.286042	-1.016743	-0.320433	0.033124	0.287309
C1 - N127	2.733714	0.275322	-0.716975	-0.427235	0.123995	0.303239
C1 - N128	2.755447	0.27042	-0.673485	-0.402157	0.116893	0.285264
C10 - H11	2.048616	0.283672	-0.998282	-0.318614	0.034522	0.284092

Atoms	distance (Å)	ρ	$\nabla^2\rho$	V	G	K
C10 - H12	2.035913	0.291113	-1.069384	-0.326008	0.029331	0.296677
C10 - N100	2.752076	0.263832	-0.66962	-0.419416	0.126005	0.29341
C10 - N98	2.732084	0.268661	-0.7096	-0.445913	0.134256	0.311657
C13 - C17	2.939989	0.243573	-0.603788	-0.257095	0.053074	0.204021
C13 - H14	2.04665	0.286165	-1.018053	-0.320517	0.033002	0.287515
C13 - N100	2.751042	0.272174	-0.67995	-0.404439	0.117226	0.287213
C13 - N99	2.735127	0.274564	-0.71605	-0.429075	0.125031	0.304044
C145 - C148	2.885758	0.248877	-0.623498	-0.273425	0.058775	0.21465
C145 - H146	2.039459	0.285891	-1.010559	-0.323849	0.035605	0.288244
C145 - H147	2.042704	0.285151	-1.00775	-0.320929	0.034496	0.286433
C145 - N155	2.83125	0.241522	-0.53756	-0.348771	0.10719	0.24158
C148 - C151	2.886102	0.248697	-0.622462	-0.274043	0.059214	0.214829
C148 - H149	2.043339	0.278034	-0.950122	-0.318123	0.040296	0.277827
C148 - H150	2.044251	0.279425	-0.957702	-0.319364	0.039969	0.279395
C15 - N101	2.623411	0.313826	-0.947416	-0.563926	0.163536	0.40039
C15 - N99	2.599126	0.321603	-1.006338	-0.606536	0.177476	0.42906
C15 - O131	2.323545	0.406687	-0.47581	-1.345308	0.613178	0.73213
C151 - H152	2.043371	0.283655	-0.993441	-0.321884	0.036762	0.285122
C151 - H153	2.048556	0.281301	-0.976904	-0.318022	0.036898	0.281124
C151 - N154	2.817776	0.246999	-0.566315	-0.355735	0.107078	0.248657
C156 - C159	2.862541	0.258443	-0.676221	-0.28854	0.059742	0.228798
C156 - H157	2.040534	0.284798	-1.001496	-0.322906	0.036266	0.28664
C156 - H158	2.039482	0.284563	-1.004482	-0.321099	0.034989	0.28611
C159 - H160	2.037945	0.286399	-1.017441	-0.323367	0.034503	0.288864
C159 - H161	2.03931	0.284717	-0.999766	-0.323996	0.037027	0.286969

Atoms	distance (Å)	ρ	$\nabla^2\rho$	V	G	K
C159 - N162	2.821634	0.246714	-0.558124	-0.351825	0.106147	0.245678
C16 - H177	6.827119	0.002139	0.008879	-0.000971	0.001595	-0.000624
C16 - N100	2.647222	0.304142	-0.880269	-0.52932	0.154626	0.374694
C16 - N102	2.636266	0.306495	-0.905217	-0.549938	0.161817	0.388121
C16 - O132	2.288073	0.424027	-0.316307	-1.468688	0.694805	0.773882
C163 - C166	2.86772	0.257224	-0.669507	-0.286048	0.059335	0.226712
C163 - H164	2.038213	0.285668	-1.005268	-0.325359	0.037021	0.288338
C163 - H165	2.037114	0.284802	-1.001277	-0.323733	0.036707	0.287026
C166 - H167	2.042599	0.283366	-0.992569	-0.321489	0.036674	0.284816
C166 - H168	2.040648	0.284984	-1.006429	-0.32139	0.034891	0.286499
C166 - N169	2.818432	0.247779	-0.564471	-0.354427	0.106655	0.247772
C17 - H18	2.047431	0.285467	-1.012819	-0.319746	0.03327	0.286475
C17 - N101	2.779911	0.262157	-0.62689	-0.383351	0.113314	0.270037
C17 - N102	2.710372	0.283977	-0.765445	-0.448657	0.128648	0.320009
C170 - C176	2.88352	0.249485	-0.626597	-0.275169	0.05926	0.215909
C170 - H171	2.042211	0.283891	-0.996192	-0.321831	0.036392	0.28544
C170 - H172	2.045597	0.283432	-0.993984	-0.319142	0.035323	0.283819
C173 - C176	2.886081	0.248768	-0.622739	-0.273909	0.059112	0.214797
C173 - H174	2.045415	0.282561	-0.982692	-0.320774	0.03755	0.283223
C173 - H175	2.04243	0.283678	-0.993163	-0.322117	0.036913	0.285204
C176 - H177	2.050654	0.275346	-0.92948	-0.316147	0.041889	0.274259
C176 - H178	2.041283	0.280891	-0.974189	-0.318591	0.037522	0.281069
C19 - H20	2.034284	0.291222	-1.065132	-0.326994	0.030355	0.296638
C19 - H21	2.057382	0.28085	-0.97626	-0.3139	0.034917	0.278982
C19 - N101	2.737657	0.269052	-0.704684	-0.433439	0.128634	0.304805

Atoms	distance (Å)	ρ	$\nabla^2\rho$	V	G	K
C19 - N103	2.743108	0.267632	-0.693958	-0.425959	0.126235	0.299724
C22 - H23	2.035831	0.29094	-1.070381	-0.325479	0.028942	0.296537
C22 - H24	2.047263	0.284692	-1.005485	-0.319958	0.034294	0.285665
C22 - N102	2.73607	0.267119	-0.697632	-0.441351	0.133471	0.307879
C22 - N104	2.739268	0.266082	-0.691192	-0.438224	0.132713	0.305511
C25 - C29	2.936419	0.244255	-0.606544	-0.258645	0.053505	0.205141
C25 - H26	2.048413	0.285089	-1.009348	-0.319382	0.033522	0.285859
C25 - N103	2.776306	0.264021	-0.635458	-0.386106	0.113621	0.272485
C25 - N104	2.711594	0.28356	-0.762476	-0.447638	0.12851	0.319129
C27 - N103	2.638718	0.308941	-0.910723	-0.538134	0.155227	0.382907
C27 - N105	2.609636	0.31689	-0.973451	-0.588851	0.172744	0.416107
C27 - O133	2.317557	0.409338	-0.443662	-1.36623	0.627657	0.738573
C28 - N104	2.633918	0.307227	-0.908897	-0.552887	0.162831	0.390056
C28 - N106	2.649901	0.303246	-0.875691	-0.526133	0.153605	0.372528
C28 - O134	2.288261	0.42396	-0.318174	-1.467974	0.694215	0.773759
C29 - H30	2.048245	0.285304	-1.011269	-0.319494	0.033339	0.286156
C29 - N105	2.73345	0.27525	-0.719596	-0.431582	0.125841	0.30574
C29 - N106	2.745989	0.274113	-0.691417	-0.40969	0.118418	0.291272
C3 - N127	2.616992	0.314205	-0.955382	-0.578336	0.169745	0.408591
C3 - N97	2.634264	0.308466	-0.913841	-0.550324	0.160932	0.389392
C3 - O129	2.300393	0.418125	-0.380361	-1.424409	0.664659	0.75975
C31 - H32	2.051275	0.283164	-0.993992	-0.31736	0.034431	0.282929
C31 - H33	2.034997	0.291097	-1.065975	-0.326372	0.029939	0.296433
C31 - N105	2.737281	0.266129	-0.696893	-0.44317	0.134473	0.308696
C31 - N107	2.73767	0.26986	-0.704673	-0.430828	0.12733	0.303498

Atoms	distance (Å)	ρ	$\nabla^2\rho$	V	G	K
C34 - H35	2.035245	0.291302	-1.071102	-0.326189	0.029207	0.296982
C34 - H36	2.050793	0.28316	-0.994206	-0.317601	0.034525	0.283076
C34 - N106	2.748908	0.265437	-0.68013	-0.421875	0.125921	0.295954
C34 - N108	2.730338	0.268733	-0.712361	-0.449412	0.135661	0.313751
C37 - C41	2.93862	0.24401	-0.606152	-0.257861	0.053162	0.2047
C37 - H38	2.047221	0.285627	-1.014167	-0.319923	0.033191	0.286732
C37 - N107	2.768627	0.265698	-0.64597	-0.391759	0.115133	0.276626
C37 - N108	2.717238	0.281066	-0.751712	-0.444076	0.128074	0.316002
C39 - H164	5.010412	0.007315	0.026171	-0.004204	0.005373	-0.001169
C39 - N107	2.642426	0.30615	-0.895276	-0.537548	0.156865	0.380684
C39 - N109	2.619598	0.313319	-0.949042	-0.573918	0.168329	0.40559
C39 - O135	2.298992	0.418641	-0.369055	-1.429661	0.668699	0.760962
C4 - H174	6.090038	0.005453	0.019696	-0.002927	0.003925	-0.000999
C4 - N128	2.63695	0.307774	-0.906279	-0.544568	0.158999	0.385569
C4 - N98	2.627711	0.310166	-0.930658	-0.563025	0.16518	0.397845
C4 - O130	2.296743	0.419882	-0.362239	-1.437489	0.673465	0.764025
C40 - N108	2.62583	0.310891	-0.935777	-0.565769	0.165912	0.399857
C40 - N110	2.64215	0.306502	-0.895177	-0.536044	0.156125	0.379919
C40 - O136	2.297958	0.419341	-0.366527	-1.433857	0.671113	0.762745
C41 - H42	2.047124	0.285831	-1.015524	-0.320118	0.033118	0.286999
C41 - N109	2.728703	0.277102	-0.727992	-0.432535	0.125268	0.307266
C41 - N110	2.761899	0.268716	-0.661067	-0.396159	0.115446	0.280713
C43 - H44	2.034751	0.291259	-1.06861	-0.326396	0.029622	0.296774
C43 - H45	2.042941	0.286233	-1.017708	-0.322158	0.033865	0.288292
C43 - N109	2.743281	0.264953	-0.682941	-0.433173	0.131219	0.301954

Atoms	distance (Å)	ρ	$\nabla^2\rho$	V	G	K
C43 - N111	2.737742	0.267083	-0.694961	-0.437833	0.132046	0.305786
C46 - H47	2.055257	0.281126	-0.978702	-0.314801	0.035063	0.279738
C46 - H48	2.036939	0.290635	-1.066185	-0.325354	0.029404	0.29595
C46 - N110	2.744325	0.266277	-0.688711	-0.428829	0.128326	0.300503
C46 - N112	2.739371	0.268541	-0.700261	-0.431637	0.128286	0.303351
C49 - C53	2.943947	0.242496	-0.599068	-0.255018	0.052626	0.202393
C49 - H50	2.04744	0.285739	-1.014504	-0.320071	0.033222	0.286848
C49 - N111	2.73236	0.275796	-0.720335	-0.429101	0.124509	0.304592
C49 - N112	2.757535	0.270113	-0.669627	-0.400007	0.1163	0.283707
C5 - H6	2.047056	0.286029	-1.016592	-0.320442	0.033147	0.287295
C5 - N97	2.761578	0.267341	-0.659565	-0.39915	0.117129	0.282021
C5 - N98	2.720628	0.280081	-0.745132	-0.439929	0.126823	0.313106
C51 - H165	5.027999	0.006261	0.021342	-0.003489	0.004412	-0.000923
C51 - N111	2.623656	0.311811	-0.939089	-0.567589	0.166408	0.40118
C51 - N113	2.632218	0.309106	-0.917272	-0.553291	0.161986	0.391304
C51 - O137	2.299824	0.418236	-0.375558	-1.426191	0.666151	0.76004
C52 - N112	2.650808	0.303371	-0.875437	-0.524158	0.152649	0.371508
C52 - N114	2.625243	0.310974	-0.93529	-0.566337	0.166257	0.40008
C52 - O138	2.293535	0.421403	-0.342981	-1.449369	0.681812	0.767557
C53 - H54	2.047137	0.285924	-1.015988	-0.320321	0.033162	0.287159
C53 - N113	2.762138	0.267202	-0.658331	-0.398848	0.117132	0.281715
C53 - N114	2.719023	0.280664	-0.747911	-0.441341	0.127182	0.314159
C55 - H56	2.035306	0.291118	-1.065892	-0.326388	0.029957	0.29643
C55 - H57	2.048315	0.284098	-1.001593	-0.318893	0.034248	0.284646
C55 - N113	2.732531	0.270644	-0.71128	-0.438582	0.130381	0.308201

Atoms	distance (Å)	ρ	$\nabla^2\rho$	V	G	K
C55 - N115	2.738811	0.265925	-0.695021	-0.441493	0.133869	0.307624
C58 - H59	2.036061	0.291042	-1.069112	-0.325807	0.029264	0.296542
C58 - H60	2.049195	0.283538	-0.997194	-0.318389	0.034545	0.283844
C58 - N114	2.737541	0.266586	-0.6975	-0.440616	0.13312	0.307495
C58 - N116	2.744237	0.266368	-0.685941	-0.427351	0.127933	0.299418
C61 - C65	2.942836	0.2428	-0.60015	-0.255651	0.052807	0.202844
C61 - H62	2.047154	0.285934	-1.016311	-0.320249	0.033086	0.287163
C61 - N115	2.735685	0.274119	-0.714005	-0.42927	0.125385	0.303886
C61 - N116	2.745425	0.273826	-0.691175	-0.410035	0.118621	0.291414
C63 - N115	2.598773	0.321577	-1.007629	-0.607719	0.177906	0.429813
C63 - N117	2.619264	0.315523	-0.958998	-0.569201	0.164726	0.404475
C63 - O139	2.324559	0.405862	-0.473145	-1.341563	0.611638	0.729924
C64 - N116	2.646142	0.303963	-0.881928	-0.532362	0.15594	0.376422
C64 - N118	2.636866	0.306483	-0.904891	-0.548647	0.161212	0.387435
C64 - O140	2.286939	0.424566	-0.310663	-1.472666	0.6975	0.775166
C65 - H66	2.047546	0.285527	-1.013098	-0.31989	0.033308	0.286582
C65 - N117	2.78117	0.261698	-0.623979	-0.3822	0.113103	0.269097
C65 - N118	2.71031	0.284122	-0.765225	-0.447914	0.128304	0.31961
C67 - H68	2.033783	0.291212	-1.063561	-0.327226	0.030668	0.296558
C67 - H69	2.056737	0.281046	-0.977966	-0.314271	0.03489	0.279381
C67 - N117	2.740812	0.26797	-0.696612	-0.429331	0.127589	0.301742
C67 - N119	2.733745	0.270335	-0.712921	-0.437995	0.129882	0.308113
C7 - H8	2.047341	0.284379	-1.003739	-0.319376	0.03422	0.285155
C7 - H9	2.035432	0.291148	-1.066838	-0.32638	0.029835	0.296545
C7 - N97	2.73239	0.270743	-0.710675	-0.438113	0.130222	0.307891

Atoms	distance (Å)	ρ	$\nabla^2\rho$	V	G	K
C7 - N99	2.741925	0.264899	-0.689022	-0.439114	0.133429	0.305685
C70 - H71	2.035967	0.290919	-1.069966	-0.325476	0.028992	0.296484
C70 - H72	2.04724	0.284541	-1.004449	-0.319904	0.034396	0.285508
C70 - N118	2.736424	0.267122	-0.69752	-0.441001	0.133311	0.307691
C70 - N120	2.740062	0.265923	-0.688814	-0.436447	0.132122	0.304325
C73 - C77	2.938314	0.243801	-0.604695	-0.257682	0.053254	0.204428
C73 - H74	2.049391	0.284839	-1.007298	-0.319039	0.033607	0.285432
C73 - N119	2.772892	0.264311	-0.64088	-0.390297	0.115038	0.275258
C73 - N120	2.71059	0.284018	-0.763878	-0.447869	0.12845	0.319419
C75 - N119	2.623158	0.313643	-0.946808	-0.56307	0.163184	0.399886
C75 - N121	2.606142	0.318424	-0.985221	-0.593661	0.173678	0.419983
C75 - O141	2.323356	0.405487	-0.452194	-1.344645	0.615798	0.728847
C76 - N120	2.637046	0.306124	-0.902139	-0.54817	0.161318	0.386852
C76 - N122	2.647801	0.303902	-0.880221	-0.529092	0.154518	0.374574
C76 - O142	2.287552	0.42429	-0.313471	-1.470725	0.696179	0.774546
C77 - H78	2.049429	0.284915	-1.007875	-0.319011	0.033521	0.28549
C77 - N121	2.738721	0.273718	-0.709382	-0.425573	0.124114	0.301459
C77 - N122	2.742671	0.275213	-0.697769	-0.412147	0.118853	0.293295
C79 - H80	2.034655	0.291117	-1.065979	-0.326476	0.029991	0.296486
C79 - H81	2.052524	0.282724	-0.990499	-0.316735	0.034555	0.28218
C79 - N121	2.735715	0.26683	-0.701014	-0.444499	0.134623	0.309876
C79 - N123	2.738286	0.26978	-0.704416	-0.429904	0.1269	0.303004
C82 - H83	2.035347	0.291231	-1.07134	-0.326039	0.029102	0.296937
C82 - H84	2.052433	0.282538	-0.98932	-0.316777	0.034724	0.282054
C82 - N122	2.748893	0.265449	-0.680794	-0.422013	0.125907	0.296106

Atoms	distance (Å)	ρ	$\nabla^2\rho$	V	G	K
C82 - N124	2.729137	0.269207	-0.714882	-0.450436	0.135858	0.314578
C85 - C89	2.938727	0.243851	-0.605397	-0.257619	0.053135	0.204484
C85 - H86	2.048592	0.285087	-1.009517	-0.319295	0.033458	0.285837
C85 - N123	2.767533	0.266083	-0.648815	-0.392948	0.115372	0.277576
C85 - N124	2.715451	0.281973	-0.755764	-0.445372	0.128216	0.317157
C87 - N123	2.637787	0.307812	-0.906817	-0.543868	0.158582	0.385286
C87 - N125	2.62106	0.312523	-0.944446	-0.571814	0.167851	0.403963
C87 - O143	2.30105	0.417632	-0.379213	-1.422352	0.663774	0.758577
C88 - N124	2.629551	0.309151	-0.923599	-0.559747	0.164424	0.395324
C88 - N126	2.649115	0.304029	-0.878989	-0.525897	0.153075	0.372822
C88 - O144	2.293248	0.421511	-0.340629	-1.450521	0.682682	0.767839
C89 - H90	2.048457	0.285321	-1.011153	-0.319552	0.033382	0.28617
C89 - N125	2.728282	0.27712	-0.72904	-0.433992	0.125866	0.308126
C89 - N126	2.757259	0.270327	-0.669987	-0.399954	0.116228	0.283725
C91 - H92	2.043334	0.286021	-1.016164	-0.321902	0.03393	0.287972
C91 - H93	2.034905	0.291217	-1.068287	-0.326349	0.029639	0.296711
C91 - N125	2.737598	0.267084	-0.695639	-0.438665	0.132378	0.306287
C91 - N127	2.741062	0.265774	-0.687471	-0.435176	0.131654	0.303522
C94 - H95	2.036913	0.290646	-1.066661	-0.325347	0.029341	0.296006
C94 - H96	2.055045	0.281184	-0.979153	-0.314957	0.035084	0.279873
C94 - N126	2.742344	0.267544	-0.693979	-0.428593	0.127549	0.301044
C94 - N128	2.740684	0.267133	-0.695113	-0.433463	0.129843	0.303621
N101 - H177	5.598504	0.004463	0.013938	-0.001979	0.002732	-0.000753
N112 - H165	5.828992	0.003722	0.012301	-0.001632	0.002354	-0.000722
N126 - H175	6.48184	0.001796	0.00657	-0.000698	0.00117	-0.000472

Atoms	distance (Å)	ρ	$\nabla^2\rho$	V	G	K
N154 - C156	2.825998	0.245761	-0.552629	-0.346738	0.10429	0.242448
N154 - Cu179	3.849683	0.085763	0.285047	-0.12243	0.096846	0.025584
N154 - H182	1.910382	0.329855	-1.645885	-0.511319	0.049924	0.461395
N155 - C163	2.816718	0.24982	-0.570057	-0.350136	0.103811	0.246325
N155 - Cu179	3.838301	0.086819	0.291203	-0.12506	0.098931	0.02613
N155 - H183	1.913304	0.328596	-1.625598	-0.508246	0.050924	0.457323
N162 - C173	2.813476	0.24878	-0.574008	-0.358036	0.107267	0.250769
N162 - Cu179	3.823994	0.087922	0.298093	-0.128076	0.1013	0.026776
N162 - H180	1.91789	0.32655	-1.605738	-0.504398	0.051482	0.452916
N169 - C170	2.820037	0.245135	-0.55974	-0.358473	0.109269	0.249204
N169 - Cu179	3.864856	0.083552	0.285352	-0.11998	0.095659	0.024321
N169 - H181	1.903148	0.334184	-1.578332	-0.503256	0.054336	0.44892
N97 - H174	5.597491	0.004451	0.01366	-0.001972	0.002693	-0.000722
O129 - H160	4.602769	0.009503	0.035751	-0.005085	0.007012	-0.001926
O130 - H178	4.933194	0.006591	0.025177	-0.003434	0.004864	-0.00143
O131 - H180	3.685192	0.026866	0.09325	-0.020889	0.022101	-0.001212
O133 - Cu179	6.566313	0.004122	0.012731	-0.002225	0.002704	-0.000479
O133 - H146	4.864026	0.006947	0.02692	-0.003775	0.005252	-0.001478
O133 - H164	5.737658	0.00347	0.012296	-0.001621	0.002347	-0.000727
O133 - H181	5.346156	0.004686	0.017355	-0.002278	0.003308	-0.001031
O134 - H172	8.202734	0.000347	0.001436	-0.000114	0.000237	-0.000122
O135 - H147	6.0678	0.003697	0.015607	-0.001857	0.002879	-0.001022
O136 - H168	5.067031	0.006651	0.024198	-0.003459	0.004754	-0.001295
O137 - H147	4.994684	0.005701	0.021338	-0.002896	0.004115	-0.001219
O138 - H168	6.45317	0.002001	0.00837	-0.000908	0.0015	-0.000592

Atoms	distance (Å)	ρ	$\nabla^2\rho$	V	G	K
O139 - H150	4.705912	0.008658	0.036204	-0.005276	0.007163	-0.001888
O139 - H183	3.769029	0.023566	0.086759	-0.017489	0.019589	-0.0021
O141 - H161	5.451712	0.00469	0.017637	-0.002383	0.003396	-0.001013
O141 - H182	3.891407	0.019034	0.077531	-0.013332	0.016357	-0.003025
O143 - H160	5.591588	0.006319	0.027057	-0.003616	0.00519	-0.001574

Table S7: Analysis of the electron density at the bond critical points for the optimized geometry at the TPSS/def2TZVP level for the *trans*-IV Cu^{II}cyclam. All values are in atomic units unless specified otherwise.

Atoms	distance (Å)	ρ	$\nabla^2\rho$	V	G	K
C1 - C5	2.932628	0.245401	-0.61209	0.024454	0.05387	0.206893
C1 - H2	2.048396	0.285026	-1.009424	0.023192	0.033394	0.28575
C1 - N127	2.749527	0.272897	-0.684626	0.033548	0.117912	0.289069
C1 - N128	2.72985	0.276756	-0.728122	0.017143	0.126261	0.308292
C10 - H11	2.057435	0.280764	-0.975558	0.032683	0.03498	0.27887
C10 - H12	2.03436	0.291193	-1.065694	0.01485	0.03021	0.296634
C10 - N100	2.739179	0.269588	-0.703215	0.04724	0.126005	0.301809
C10 - N98	2.748199	0.26506	-0.682209	0.053614	0.1267	0.297252
C13 - C17	2.942657	0.242784	-0.600347	0.024043	0.052753	0.202839
C13 - H14	2.048608	0.285083	-1.00934	0.024179	0.033499	0.285834
C13 - N100	2.776916	0.263657	-0.632708	0.024343	0.112899	0.271076
C13 - N99	2.713623	0.282516	-0.758572	0.005736	0.128462	0.318105
C149 - C153	2.87898	0.254594	-0.655899	0.043926	0.058361	0.222336
C149 - H150	2.037321	0.286458	-1.019596	0.021625	0.034054	0.288953

Atoms	distance (Å)	ρ	$\nabla^2\rho$	V	G	K
C149 - H151	2.039336	0.284514	-0.995125	0.027762	0.038049	0.28683
C15 - N101	2.641585	0.305742	-0.893616	0.198098	0.15753	0.380934
C15 - N99	2.634284	0.307216	-0.910513	0.201135	0.162708	0.390337
C15 - O131	2.289266	0.423414	-0.322152	0.13861	0.691831	0.77237
C153 - H154	2.037705	0.2855	-1.00712	0.023083	0.036192	0.287972
C155 - C176	2.907543	0.242711	-0.593421	0.027859	0.057191	0.205546
C155 - H156	2.042687	0.284909	-1.007176	0.021739	0.034238	0.286032
C155 - H157	2.044445	0.282636	-0.984788	0.029901	0.037873	0.28407
C158 - C176	2.907566	0.242706	-0.593395	0.027861	0.057189	0.205538
C158 - H159	2.042681	0.284911	-1.007196	0.021737	0.034237	0.286036
C158 - H160	2.04445	0.282635	-0.984778	0.029902	0.037873	0.284067
C16 - N100	2.642983	0.306841	-0.898199	0.196843	0.154661	0.379211
C16 - N102	2.609761	0.317005	-0.976021	0.213649	0.173266	0.417271
C16 - O132	2.306127	0.415103	-0.400379	0.131984	0.652546	0.75264
C162 - C163	2.87897	0.254597	-0.655912	0.043927	0.058362	0.22234
C162 - H183	2.042892	0.282992	-0.988732	0.027217	0.037196	0.284379
C163 - H164	2.039324	0.284518	-0.995155	0.02776	0.038048	0.286837
C163 - H165	2.03732	0.28646	-1.019609	0.021625	0.034052	0.288955
C166 - C173	2.899433	0.244984	-0.60474	0.026962	0.057638	0.208823
C166 - H167	2.042512	0.284151	-0.997413	0.023163	0.035913	0.285267
C166 - H171	2.036206	0.287483	-1.017485	0.026729	0.036467	0.290838
C168 - C173	2.89945	0.24498	-0.60472	0.026964	0.057636	0.208816
C168 - H169	2.042506	0.284153	-0.997427	0.02316	0.035913	0.28527
C168 - H170	2.036196	0.287485	-1.017501	0.02673	0.036469	0.290844
C17 - H18	2.047578	0.285913	-1.015558	0.025105	0.033193	0.287083

Atoms	distance (Å)	ρ	$\nabla^2\rho$	V	G	K
C17 - N101	2.7514	0.271395	-0.679201	0.037716	0.11796	0.28776
C17 - N102	2.726919	0.27737	-0.732434	0.002359	0.126791	0.309899
C173 - H174	2.042453	0.2797	-0.96001	0.008127	0.040047	0.28005
C176 - H177	2.04092	0.280813	-0.969456	0.010725	0.038942	0.281306
C19 - H20	2.035787	0.291145	-1.06825	0.019407	0.029531	0.296593
C19 - H21	2.04706	0.28423	-1.002677	0.035984	0.034381	0.28505
C19 - N101	2.740895	0.267493	-0.691733	0.044791	0.128745	0.301678
C19 - N103	2.740252	0.266045	-0.694256	0.043131	0.132108	0.305672
C22 - H23	2.035767	0.291078	-1.066939	0.019297	0.029727	0.296462
C22 - H24	2.047064	0.284297	-1.003154	0.035537	0.034331	0.28512
C22 - N102	2.737029	0.26704	-0.70056	0.041083	0.133506	0.308646
C22 - N104	2.73954	0.267928	-0.694511	0.045378	0.129204	0.302832
C25 - C29	2.945145	0.242331	-0.598507	0.022686	0.05248	0.202107
C25 - H26	2.046023	0.286521	-1.020613	0.025299	0.032942	0.288095
C25 - N103	2.726815	0.277944	-0.732283	0.003379	0.125184	0.308255
C25 - N104	2.76193	0.267507	-0.657746	0.039628	0.116369	0.280806
C27 - N103	2.617997	0.314536	-0.958354	0.20765	0.168494	0.408082
C27 - N105	2.641054	0.3062	-0.897125	0.198994	0.158104	0.382385
C27 - O133	2.299763	0.4183	-0.374109	0.134829	0.666758	0.760285
C28 - N104	2.634744	0.308331	-0.910345	0.20156	0.16058	0.388166
C28 - N106	2.628451	0.309955	-0.928109	0.203095	0.164632	0.396659
C28 - O134	2.295454	0.420495	-0.355642	0.134866	0.67652	0.765431
C29 - H30	2.046744	0.285983	-1.016785	0.024901	0.033078	0.287274
C29 - N105	2.762961	0.267422	-0.657971	0.035612	0.116297	0.28079
C29 - N106	2.725631	0.278147	-0.733336	0.00333	0.12561	0.308944

Atoms	distance (Å)	ρ	$\nabla^2\rho$	V	G	K
C3 - N127	2.650403	0.303232	-0.876385	0.191812	0.153628	0.372724
C3 - N97	2.628889	0.309383	-0.922994	0.205612	0.164628	0.395377
C3 - O129	2.290818	0.422704	-0.330832	0.139622	0.688009	0.770717
C31 - H32	2.054485	0.281561	-0.982011	0.033922	0.034882	0.280385
C31 - H33	2.036386	0.290728	-1.064984	0.015433	0.029725	0.295971
C31 - N105	2.728948	0.271711	-0.721132	0.049751	0.131782	0.312065
C31 - N107	2.753075	0.263406	-0.669776	0.044419	0.125544	0.292989
C34 - H35	2.034936	0.291264	-1.069382	0.020906	0.029468	0.296814
C34 - H36	2.042838	0.28621	-1.017532	0.038023	0.033907	0.28829
C34 - N106	2.737557	0.267001	-0.694266	0.033278	0.132028	0.305595
C34 - N108	2.745352	0.264307	-0.678962	0.04094	0.130789	0.300529
C37 - C41	2.93818	0.243989	-0.605399	0.024159	0.053341	0.204691
C37 - H38	2.046495	0.286027	-1.017009	0.024312	0.033109	0.287361
C37 - N107	2.769296	0.266365	-0.647812	0.021836	0.114446	0.2764
C37 - N108	2.722914	0.279516	-0.740215	0.004729	0.125968	0.311021
C39 - N107	2.637743	0.308932	-0.910441	0.202098	0.156419	0.384029
C39 - N109	2.616556	0.314562	-0.959746	0.212559	0.169454	0.409391
C39 - O135	2.31008	0.413401	-0.421399	0.129542	0.643291	0.748641
C4 - N128	2.612758	0.315954	-0.969103	0.217925	0.170649	0.412925
C4 - N98	2.627831	0.312434	-0.934662	0.21118	0.160314	0.39398
C4 - O130	2.321712	0.407063	-0.458043	0.125115	0.618478	0.732989
C40 - N108	2.626228	0.310894	-0.931575	0.206878	0.165167	0.39806
C40 - N110	2.647596	0.304182	-0.882504	0.193322	0.15513	0.375756
C40 - O136	2.29371	0.421257	-0.343938	0.138709	0.681188	0.767173
C41 - H42	2.046937	0.285612	-1.01422	0.023723	0.033153	0.286708

Atoms	distance (Å)	ρ	$\nabla^2\rho$	V	G	K
C41 - N109	2.722896	0.279032	-0.74078	0.017901	0.127476	0.312671
C41 - N110	2.761528	0.268415	-0.660055	0.037007	0.116014	0.281028
C43 - H44	2.034594	0.291441	-1.07096	0.016992	0.029386	0.297126
C43 - H45	2.052531	0.282791	-0.991289	0.034723	0.034474	0.282296
C43 - N109	2.732977	0.26722	-0.704895	0.037872	0.135993	0.312217
C43 - N111	2.742167	0.268353	-0.697314	0.069161	0.12621	0.300539
C46 - H47	2.05224	0.282998	-0.992526	0.034444	0.034425	0.282556
C46 - H48	2.034801	0.291206	-1.06819	0.017144	0.029644	0.296692
C46 - N110	2.74327	0.268089	-0.695066	0.068957	0.12567	0.299436
C46 - N112	2.735343	0.266585	-0.700374	0.036975	0.134828	0.309921
C49 - C53	2.932648	0.245396	-0.612062	0.024455	0.053868	0.206884
C49 - H50	2.048395	0.285026	-1.009424	0.023188	0.033395	0.285751
C49 - N111	2.749486	0.272908	-0.684708	0.03356	0.117924	0.289101
C49 - N112	2.729893	0.276745	-0.72804	0.017156	0.126245	0.308255
C5 - H6	2.047623	0.285419	-1.012281	0.023775	0.033332	0.286402
C5 - N97	2.71309	0.28294	-0.758832	0.008823	0.128272	0.31798
C5 - N98	2.777101	0.263721	-0.63345	0.02448	0.113357	0.271719
C51 - N111	2.650362	0.303242	-0.876469	0.191832	0.153651	0.372768
C51 - N113	2.628898	0.309379	-0.922971	0.205607	0.164624	0.395367
C51 - O137	2.290818	0.422704	-0.330849	0.139624	0.688007	0.770719
C52 - N112	2.612744	0.315959	-0.969141	0.217912	0.170655	0.41294
C52 - N114	2.627801	0.312441	-0.934722	0.211208	0.160333	0.394013
C52 - O138	2.321704	0.407067	-0.458017	0.125115	0.618494	0.732998
C53 - H54	2.047624	0.285419	-1.012282	0.023773	0.033332	0.286402
C53 - N113	2.713076	0.282945	-0.758863	0.008829	0.128275	0.317991

Atoms	distance (Å)	ρ	$\nabla^2\rho$	V	G	K
C53 - N114	2.777109	0.263717	-0.633433	0.024484	0.113356	0.271714
C55 - H56	2.035692	0.290971	-1.069578	0.018661	0.029112	0.296506
C55 - H57	2.046754	0.284828	-1.00667	0.038502	0.034275	0.285942
C55 - N113	2.741436	0.264992	-0.685259	0.042884	0.13299	0.304305
C55 - N115	2.728956	0.269914	-0.713938	0.042834	0.134924	0.313409
C58 - H59	2.034361	0.291193	-1.065692	0.014849	0.03021	0.296633
C58 - H60	2.057431	0.280765	-0.975567	0.032683	0.03498	0.278871
C58 - N114	2.748198	0.265059	-0.682205	0.053607	0.126704	0.297256
C58 - N116	2.739193	0.269586	-0.703196	0.047236	0.125997	0.301796
C61 - C65	2.942618	0.242795	-0.6004	0.024037	0.052756	0.202856
C61 - H62	2.048606	0.285083	-1.009345	0.024182	0.033499	0.285835
C61 - N115	2.713623	0.282515	-0.758569	0.005728	0.128462	0.318104
C61 - N116	2.776965	0.263644	-0.632615	0.024346	0.112886	0.27104
C63 - N115	2.634299	0.307213	-0.910486	0.201134	0.162699	0.390321
C63 - N117	2.641608	0.305737	-0.893574	0.19808	0.157515	0.380909
C63 - O139	2.289259	0.423416	-0.322097	0.138608	0.691851	0.772375
C64 - N116	2.643027	0.306829	-0.898101	0.196825	0.154636	0.379162
C64 - N118	2.609766	0.317005	-0.97602	0.213643	0.173261	0.417267
C64 - O140	2.306127	0.415103	-0.400351	0.131984	0.652551	0.752639
C65 - H66	2.047576	0.285913	-1.01556	0.025104	0.033193	0.287083
C65 - N117	2.75143	0.271388	-0.679143	0.037714	0.117951	0.287737
C65 - N118	2.726899	0.277376	-0.732477	0.002353	0.126797	0.309916
C67 - H68	2.035782	0.291147	-1.06826	0.019406	0.029532	0.296597
C67 - H69	2.047072	0.284226	-1.002647	0.035984	0.034381	0.285043
C67 - N117	2.740907	0.267493	-0.691718	0.044791	0.128737	0.301667

Atoms	distance (Å)	ρ	$\nabla^2\rho$	V	G	K
C67 - N119	2.740248	0.266046	-0.694262	0.04313	0.13211	0.305675
C7 - H8	2.046763	0.284825	-1.006649	0.038502	0.034275	0.285937
C7 - H9	2.035692	0.290971	-1.069578	0.018659	0.029112	0.296506
C7 - N97	2.741435	0.264992	-0.685267	0.042899	0.132991	0.304308
C7 - N99	2.728944	0.269917	-0.713956	0.042836	0.134929	0.313418
C70 - H71	2.035765	0.291079	-1.066944	0.019298	0.029728	0.296464
C70 - H72	2.047066	0.284297	-1.00315	0.035537	0.034331	0.285119
C70 - N118	2.737029	0.267041	-0.70057	0.04109	0.133505	0.308647
C70 - N120	2.739547	0.267928	-0.694501	0.045367	0.1292	0.302825
C73 - C77	2.945144	0.242331	-0.598506	0.022687	0.05248	0.202107
C73 - H74	2.046021	0.286521	-1.020614	0.025297	0.032942	0.288096
C73 - N119	2.726812	0.277947	-0.732293	0.003389	0.125183	0.308256
C73 - N120	2.761951	0.267502	-0.657709	0.03961	0.116363	0.28079
C75 - N119	2.618049	0.314519	-0.95822	0.207648	0.168463	0.408018
C75 - N121	2.641016	0.306211	-0.897217	0.199	0.158123	0.382428
C75 - O141	2.299754	0.418304	-0.374061	0.134827	0.666778	0.760294
C76 - N120	2.63477	0.308325	-0.91029	0.201548	0.160564	0.388137
C76 - N122	2.628443	0.309957	-0.928117	0.203098	0.164638	0.396667
C76 - O142	2.295416	0.420512	-0.355423	0.13486	0.676614	0.765469
C77 - H78	2.046744	0.285983	-1.016784	0.024901	0.033078	0.287274
C77 - N121	2.76294	0.267428	-0.658012	0.035617	0.116303	0.280806
C77 - N122	2.725636	0.278145	-0.733324	0.003331	0.12561	0.308941
C79 - H80	2.036385	0.290728	-1.064989	0.015432	0.029725	0.295972
C79 - H81	2.054491	0.281559	-0.981995	0.033923	0.034882	0.280381
C79 - N121	2.728934	0.271715	-0.721159	0.049759	0.131789	0.312079

Atoms	distance (Å)	ρ	$\nabla^2\rho$	V	G	K
C79 - N123	2.753077	0.263407	-0.669775	0.044422	0.125542	0.292985
C82 - H83	2.03494	0.291263	-1.069373	0.020907	0.029468	0.296811
C82 - H84	2.042835	0.286211	-1.017539	0.038022	0.033907	0.288292
C82 - N122	2.737566	0.266999	-0.694252	0.033272	0.132025	0.305588
C82 - N124	2.745348	0.264309	-0.678977	0.040945	0.130791	0.300535
C85 - C89	2.938148	0.243998	-0.605442	0.024158	0.053344	0.204704
C85 - H86	2.046492	0.286028	-1.017013	0.024311	0.033109	0.287362
C85 - N123	2.769299	0.266366	-0.647808	0.021836	0.114444	0.276396
C85 - N124	2.722917	0.279515	-0.740213	0.004722	0.125968	0.311021
C87 - N123	2.637769	0.308925	-0.910391	0.202078	0.156405	0.384003
C87 - N125	2.616562	0.314561	-0.959731	0.212561	0.169451	0.409384
C87 - O143	2.310101	0.413391	-0.421502	0.129549	0.643243	0.748619
C88 - N124	2.626206	0.310902	-0.931632	0.206882	0.165178	0.398086
C88 - N126	2.647625	0.304175	-0.882446	0.193305	0.155112	0.375724
C88 - O144	2.293726	0.42125	-0.344013	0.138713	0.681153	0.767156
C89 - H90	2.046934	0.285613	-1.014227	0.023725	0.033153	0.286709
C89 - N125	2.722893	0.279032	-0.740783	0.017888	0.127478	0.312673
C89 - N126	2.761569	0.268404	-0.659974	0.036994	0.116003	0.280996
C91 - H92	2.052527	0.282792	-0.991295	0.034723	0.034474	0.282298
C91 - H93	2.034593	0.291442	-1.070963	0.016995	0.029386	0.297127
C91 - N125	2.732964	0.267225	-0.704926	0.037887	0.136	0.312231
C91 - N127	2.742186	0.268349	-0.697272	0.069133	0.126202	0.30052
C94 - H95	2.034798	0.291208	-1.068201	0.017147	0.029644	0.296694
C94 - H96	2.05224	0.282999	-0.992527	0.034445	0.034425	0.282556
C94 - N126	2.743296	0.268082	-0.695008	0.068935	0.12566	0.299412

Atoms	distance (Å)	ρ	$\nabla^2\rho$	V	G	K
C94 - N128	2.735323	0.266591	-0.700416	0.036988	0.134838	0.309942
H152 - C153	2.042892	0.282992	-0.988731	0.027215	0.037196	0.284378
H161 - C162	2.037711	0.285497	-1.007103	0.023087	0.036191	0.287967
H172 - C173	2.042452	0.2797	-0.960008	0.008128	0.040048	0.28005
H175 - C176	2.040932	0.280808	-0.969421	0.010725	0.038944	0.281299
N105 - H154	4.881263	0.009855	0.0319	0.691089	0.006583	-0.001392
N107 - H151	6.041922	0.003138	0.010955	0.591019	0.002045	-0.000694
N110 - H151	5.220627	0.007548	0.024954	1.208541	0.005057	-0.001182
N121 - H161	4.881219	0.009854	0.031895	0.689881	0.006582	-0.001392
N123 - H164	6.042931	0.003135	0.010945	0.597897	0.002043	-0.000694
N126 - H164	5.220675	0.00755	0.024964	1.212314	0.005059	-0.001182
N145 - C153	2.816204	0.248435	-0.567728	0.029457	0.106755	0.248687
N145 - C155	2.817355	0.245667	-0.563337	0.035732	0.110237	0.251071
N145 - Cu178	3.824466	0.087633	0.303256	0.029933	0.102129	0.026315
N145 - H182	1.903585	0.333093	-1.623403	0.021711	0.051847	0.457698
N146 - C158	2.817358	0.245666	-0.563331	0.035733	0.110237	0.25107
N146 - C162	2.816257	0.248421	-0.567643	0.029449	0.106743	0.248653
N146 - Cu178	3.8244	0.087639	0.303289	0.029937	0.102141	0.026319
N146 - H179	1.903592	0.33309	-1.623327	0.021714	0.051848	0.45768
N147 - C149	2.831163	0.244923	-0.546488	0.021354	0.101346	0.237968
N147 - C168	2.822342	0.244668	-0.556677	0.037654	0.108013	0.247182
N147 - Cu178	3.832021	0.087491	0.293408	0.02966	0.099802	0.02645
N147 - H181	1.911091	0.328961	-1.661359	0.023548	0.04979	0.46513
N148 - C163	2.831197	0.244913	-0.546433	0.021351	0.10134	0.237949
N148 - C166	2.822355	0.244665	-0.556659	0.037653	0.108009	0.247174

Atoms	distance (Å)	ρ	$\nabla^2\rho$	V	G	K
N148 - Cu178	3.831929	0.0875	0.293449	0.029658	0.099817	0.026455
N148 - H180	1.91113	0.328945	-1.661151	0.023547	0.049787	0.465075
O129 - H157	7.557868	0.000532	0.002115	0.682919	0.000353	-0.000176
O130 - H170	5.408299	0.004493	0.016269	0.292698	0.00315	-0.000918
O130 - H180	3.77264	0.022605	0.083741	0.046568	0.018619	-0.002317
O132 - H150	5.510498	0.004274	0.016922	0.155034	0.0032	-0.001031
O132 - H152	5.651089	0.004248	0.016404	0.912223	0.003141	-0.00096
O132 - H170	4.836755	0.008798	0.0347	0.192022	0.006935	-0.001739
O133 - H156	5.112203	0.005149	0.01895	0.0138	0.003653	-0.001084
O134 - H150	5.108251	0.005775	0.021812	0.290924	0.004167	-0.001286
O134 - O136	6.293943	0.004353	0.01724	3.791464	0.003196	-0.001114
O135 - H175	5.740324	0.003039	0.013	0.510582	0.002388	-0.000862
O135 - H182	4.473164	0.010666	0.040907	0.046955	0.007984	-0.002243
O137 - H160	7.559487	0.000532	0.002112	0.682778	0.000352	-0.000176
O138 - H171	5.407213	0.004498	0.016287	0.291912	0.003153	-0.000918
O138 - H181	3.772544	0.022609	0.083751	0.046577	0.018622	-0.002316
O140 - H165	5.50995	0.004276	0.016928	0.155052	0.003201	-0.001031
O140 - H171	4.837368	0.008793	0.034676	0.192016	0.006931	-0.001739
O140 - H183	5.650947	0.004247	0.016401	0.911277	0.00314	-0.00096
O141 - H159	5.111259	0.005155	0.018969	0.013839	0.003657	-0.001085
O142 - H165	5.107752	0.005778	0.021824	0.290567	0.004169	-0.001287
O142 - O144	6.294413	0.004352	0.017235	3.845956	0.003195	-0.001114
O143 - H177	5.738768	0.003043	0.013017	0.507871	0.002392	-0.000863
O143 - H179	4.473701	0.01066	0.040888	0.047191	0.00798	-0.002242

Table S8: Analysis of the electron density at the bond critical points for the optimized geometry at the TPSS/def2TZVP level for the *trans*-V Cu^{II}cyclam. All values are in atomic units unless specified otherwise.

Atoms	distance (Å)	ρ	$\nabla^2\rho$	V	G	K
C1 - C5	2.931662	0.245906	-0.615358	-0.261464	0.053812	0.207652
C1 - H2	2.047978	0.285253	-1.011358	-0.319421	0.033291	0.28613
C1 - N127	2.760305	0.269267	-0.662547	-0.39702	0.115692	0.281329
C1 - N128	2.724347	0.278404	-0.737907	-0.438732	0.127128	0.311604
C10 - H11	2.056848	0.280987	-0.977258	-0.314232	0.034959	0.279273
C10 - H12	2.034479	0.291138	-1.065445	-0.32671	0.030174	0.296536
C10 - N100	2.74798	0.265068	-0.681514	-0.423561	0.126591	0.29697
C10 - N98	2.735959	0.270272	-0.710046	-0.433199	0.127844	0.305355
C13 - C17	2.94581	0.241686	-0.594353	-0.25379	0.052601	0.201189
C13 - H14	2.04887	0.285224	-1.009847	-0.319574	0.033556	0.286018
C13 - N100	2.771311	0.265228	-0.644945	-0.390519	0.114642	0.275878
C13 - N99	2.714742	0.282457	-0.755064	-0.444	0.127617	0.316383
C15 - N101	2.641039	0.305499	-0.894609	-0.540464	0.158406	0.382058
C15 - N99	2.631849	0.30787	-0.913967	-0.556222	0.163865	0.392357
C15 - O131	2.288985	0.423625	-0.323549	-1.465006	0.692059	0.772946
C150 - C153	2.871826	0.256155	-0.663522	-0.283991	0.059055	0.224936
C150 - H151	2.034795	0.286935	-1.017074	-0.326035	0.035883	0.290152
C150 - H152	2.040328	0.284037	-0.994382	-0.32359	0.037497	0.286092
C153 - H154	2.038971	0.286147	-1.016593	-0.323033	0.034443	0.288591
C153 - H155	2.043044	0.283133	-0.989342	-0.321616	0.03714	0.284476
C156 - C159	2.872208	0.255905	-0.661982	-0.283847	0.059176	0.224671
C156 - H157	2.04134	0.284698	-1.001005	-0.322684	0.036216	0.286468
C156 - H158	2.039125	0.286176	-1.018455	-0.322649	0.034018	0.288632

Atoms	distance (Å)	ρ	$\nabla^2\rho$	V	G	K
C159 - H160	2.041698	0.283645	-0.992783	-0.32254	0.037172	0.285368
C159 - H161	2.03947	0.285096	-1.004733	-0.323191	0.036004	0.287187
C16 - N100	2.624833	0.313437	-0.943541	-0.558813	0.161464	0.397349
C16 - N102	2.609284	0.317028	-0.975744	-0.589246	0.172655	0.416591
C16 - O132	2.322847	0.406086	-0.456402	-1.346816	0.616358	0.730458
C162 - C165	2.902336	0.244125	-0.600962	-0.264742	0.057251	0.207491
C162 - H163	2.045024	0.282772	-0.987619	-0.319655	0.036375	0.28328
C162 - H164	2.037535	0.287419	-1.018302	-0.326552	0.035988	0.290564
C165 - C168	2.898642	0.244949	-0.604703	-0.266534	0.057679	0.208855
C165 - H166	2.042662	0.279639	-0.959183	-0.320218	0.040211	0.280007
C165 - H167	2.042391	0.279802	-0.960326	-0.320343	0.040131	0.280212
C168 - H169	2.036729	0.287667	-1.019609	-0.327056	0.036077	0.290979
C168 - H170	2.044446	0.28321	-0.991313	-0.319773	0.035972	0.283801
C17 - H18	2.048995	0.285245	-1.010207	-0.319486	0.033467	0.286019
C17 - N101	2.736969	0.276344	-0.709453	-0.419284	0.12096	0.298323
C17 - N102	2.737992	0.274082	-0.711069	-0.426542	0.124388	0.302155
C171 - C177	2.903125	0.243745	-0.598012	-0.264781	0.057639	0.207142
C171 - H172	2.044017	0.282933	-0.986837	-0.322325	0.037808	0.284517
C171 - H173	2.040246	0.286256	-1.016181	-0.322012	0.033983	0.288028
C174 - C177	2.906459	0.242981	-0.594723	-0.263204	0.057262	0.205943
C174 - H175	2.045339	0.282385	-0.982887	-0.321977	0.038128	0.283849
C174 - H176	2.042775	0.285173	-1.008503	-0.320542	0.034208	0.286334
C177 - H178	2.042119	0.280032	-0.963628	-0.319611	0.039352	0.280259
C177 - H179	2.042322	0.280508	-0.968149	-0.319105	0.038534	0.280571
C19 - H20	2.035604	0.291166	-1.069822	-0.326015	0.02928	0.296735

Atoms	distance (Å)	ρ	$\nabla^2\rho$	V	G	K
C19 - H21	2.049449	0.283588	-0.997552	-0.318355	0.034484	0.283872
C19 - N101	2.7406	0.267451	-0.693607	-0.431918	0.129258	0.30266
C19 - N103	2.734144	0.267554	-0.704454	-0.445376	0.134631	0.310745
C22 - H23	2.035297	0.291038	-1.066202	-0.32626	0.029855	0.296405
C22 - H24	2.051322	0.283072	-0.993305	-0.317328	0.034501	0.282827
C22 - N102	2.737453	0.266452	-0.697156	-0.441605	0.133658	0.307947
C22 - N104	2.739333	0.268795	-0.699592	-0.430421	0.127761	0.302659
C25 - C29	2.939864	0.243781	-0.605373	-0.257322	0.05299	0.204333
C25 - H26	2.046593	0.285992	-1.016977	-0.320354	0.033055	0.287299
C25 - N103	2.717194	0.28125	-0.752084	-0.44335	0.127664	0.315685
C25 - N104	2.771705	0.264542	-0.63947	-0.388833	0.114483	0.27435
C27 - N103	2.624837	0.311462	-0.9391	-0.566685	0.165955	0.40073
C27 - N105	2.642926	0.305656	-0.892218	-0.536754	0.15685	0.379904
C27 - O133	2.296111	0.420075	-0.355685	-1.43987	0.675474	0.764396
C28 - N104	2.633569	0.309171	-0.915452	-0.550437	0.160787	0.38965
C28 - N106	2.621121	0.312977	-0.947686	-0.571934	0.167506	0.404428
C28 - O134	2.301573	0.41754	-0.385651	-1.420355	0.661971	0.758384
C29 - H30	2.046634	0.286053	-1.017324	-0.320406	0.033037	0.287368
C29 - N105	2.761146	0.26833	-0.661019	-0.397597	0.116171	0.281426
C29 - N106	2.728744	0.277049	-0.727625	-0.432291	0.125193	0.307099
C3 - N127	2.649714	0.303769	-0.877485	-0.526022	0.153325	0.372696
C3 - N97	2.630264	0.309041	-0.922366	-0.558749	0.164079	0.39467
C3 - O129	2.291517	0.422318	-0.332027	-1.4565	0.686747	0.769753
C31 - H32	2.053215	0.281913	-0.98484	-0.315894	0.034842	0.281052
C31 - H33	2.036684	0.290621	-1.064988	-0.325441	0.029597	0.295844

Atoms	distance (Å)	ρ	$\nabla^2\rho$	V	G	K
C31 - N105	2.735026	0.269623	-0.707702	-0.437346	0.13021	0.307136
C31 - N107	2.745656	0.265505	-0.684273	-0.427876	0.128404	0.299472
C34 - H35	2.034975	0.291272	-1.069106	-0.326345	0.029535	0.296811
C34 - H36	2.042666	0.286208	-1.017689	-0.322185	0.033881	0.288304
C34 - N106	2.742587	0.265314	-0.685154	-0.433404	0.131058	0.302346
C34 - N108	2.739982	0.266238	-0.689476	-0.435873	0.131752	0.304121
C37 - C41	2.949341	0.240943	-0.591203	-0.252216	0.052208	0.200009
C37 - H38	2.047199	0.286032	-1.016358	-0.320468	0.033189	0.287279
C37 - N107	2.758125	0.269276	-0.668847	-0.401427	0.117108	0.28432
C37 - N108	2.727582	0.277664	-0.729568	-0.432663	0.125136	0.307528
C39 - N107	2.635444	0.308647	-0.912792	-0.546171	0.158987	0.387185
C39 - N109	2.618916	0.313317	-0.950766	-0.575598	0.168953	0.406645
C39 - O135	2.303433	0.416578	-0.392817	-1.414165	0.65798	0.756185
C4 - N128	2.616217	0.314639	-0.960518	-0.57959	0.16973	0.40986
C4 - N98	2.638162	0.308181	-0.907374	-0.541587	0.157372	0.384215
C4 - O130	2.306067	0.415074	-0.40018	-1.405039	0.652497	0.752542
C40 - N108	2.626172	0.31042	-0.929681	-0.564164	0.165872	0.398292
C40 - N110	2.641553	0.306065	-0.895838	-0.538562	0.157301	0.381261
C40 - O136	2.293713	0.421262	-0.344993	-1.448181	0.680966	0.767214
C41 - H42	2.047737	0.28568	-1.013997	-0.319998	0.033249	0.286749
C41 - N109	2.727229	0.277637	-0.731665	-0.434891	0.125987	0.308903
C41 - N110	2.754826	0.27035	-0.673979	-0.403431	0.117468	0.285963
C43 - H44	2.035421	0.291181	-1.069222	-0.326178	0.029436	0.296742
C43 - H45	2.051126	0.28297	-0.992786	-0.317325	0.034564	0.282761
C43 - N109	2.730349	0.268782	-0.712172	-0.449873	0.135915	0.313958

Atoms	distance (Å)	ρ	$\nabla^2\rho$	V	G	K
C43 - N111	2.750936	0.265242	-0.676212	-0.417955	0.124451	0.293504
C46 - H47	2.049355	0.283701	-0.998292	-0.318386	0.034406	0.283979
C46 - H48	2.035377	0.291159	-1.068497	-0.326223	0.029549	0.296673
C46 - N110	2.741865	0.267817	-0.692809	-0.428203	0.1275	0.300703
C46 - N112	2.734472	0.267331	-0.704082	-0.446545	0.135262	0.311283
C49 - C53	2.931348	0.245863	-0.614836	-0.261484	0.053887	0.207597
C49 - H50	2.047293	0.28556	-1.013654	-0.319821	0.033204	0.286617
C49 - N111	2.761899	0.269053	-0.660428	-0.395424	0.115158	0.280265
C49 - N112	2.723821	0.278594	-0.739383	-0.439777	0.127466	0.312311
C5 - H6	2.047865	0.285226	-1.011085	-0.319461	0.033345	0.286116
C5 - N97	2.712608	0.282824	-0.760748	-0.448023	0.128918	0.319105
C5 - N98	2.776392	0.263604	-0.632105	-0.384744	0.113359	0.271385
C51 - N111	2.652825	0.303034	-0.871068	-0.520565	0.151399	0.369166
C51 - N113	2.628963	0.309375	-0.923962	-0.560551	0.16478	0.395771
C51 - O137	2.292532	0.421829	-0.337152	-1.452985	0.684348	0.768636
C52 - N112	2.613932	0.315548	-0.965626	-0.583006	0.1708	0.412206
C52 - N114	2.640573	0.307717	-0.903744	-0.537484	0.155774	0.38171
C52 - O138	2.307619	0.414327	-0.406681	-1.399874	0.649102	0.750772
C53 - H54	2.047631	0.285272	-1.01143	-0.319515	0.033329	0.286186
C53 - N113	2.713043	0.282722	-0.760314	-0.447992	0.128957	0.319035
C53 - N114	2.776978	0.26373	-0.632034	-0.384097	0.113044	0.271053
C55 - H56	2.035685	0.291003	-1.07018	-0.325642	0.029048	0.296594
C55 - H57	2.048325	0.284282	-1.002494	-0.319302	0.034339	0.284963
C55 - N113	2.734351	0.267547	-0.702228	-0.444622	0.134532	0.310089
C55 - N115	2.735671	0.267676	-0.699748	-0.441134	0.133099	0.308036

Atoms	distance (Å)	ρ	$\nabla^2\rho$	V	G	K
C58 - H59	2.034194	0.291268	-1.066607	-0.326882	0.030115	0.296767
C58 - H60	2.056983	0.28102	-0.97752	-0.314163	0.034891	0.279271
C58 - N114	2.740628	0.268849	-0.700739	-0.427817	0.126316	0.301501
C58 - N116	2.743392	0.266482	-0.691267	-0.428986	0.128085	0.300901
C61 - C65	2.948136	0.241051	-0.591229	-0.25261	0.052402	0.200209
C61 - H62	2.04903	0.285129	-1.009162	-0.319397	0.033553	0.285844
C61 - N115	2.717074	0.281881	-0.7508	-0.441378	0.126839	0.314539
C61 - N116	2.767305	0.266391	-0.653371	-0.394559	0.115608	0.278951
C63 - N115	2.633223	0.307355	-0.910311	-0.554017	0.16322	0.390797
C63 - N117	2.638572	0.30621	-0.899414	-0.544002	0.159574	0.384428
C63 - O139	2.289284	0.423512	-0.32609	-1.463875	0.691176	0.772699
C64 - N116	2.624166	0.313389	-0.944698	-0.560794	0.16231	0.398484
C64 - N118	2.606493	0.318011	-0.981477	-0.593856	0.174244	0.419613
C64 - O140	2.322461	0.406439	-0.459205	-1.347961	0.61658	0.731381
C65 - H66	2.048553	0.28556	-1.012526	-0.319857	0.033363	0.286494
C65 - N117	2.734457	0.276944	-0.71448	-0.422205	0.121792	0.300412
C65 - N118	2.740514	0.273296	-0.705763	-0.423694	0.123627	0.300068
C67 - H68	2.035527	0.291227	-1.06971	-0.326159	0.029366	0.296793
C67 - H69	2.047743	0.284146	-1.002002	-0.319206	0.034353	0.284853
C67 - N117	2.741352	0.266723	-0.689792	-0.432481	0.130017	0.302465
C67 - N119	2.733675	0.268079	-0.706518	-0.44478	0.134075	0.310705
C7 - H8	2.047115	0.284645	-1.005335	-0.319976	0.034321	0.285655
C7 - H9	2.035695	0.291003	-1.069564	-0.32568	0.029145	0.296536
C7 - N97	2.734302	0.267651	-0.701829	-0.443902	0.134223	0.30968
C7 - N99	2.735486	0.267571	-0.699347	-0.44202	0.133592	0.308428

Atoms	distance (Å)	ρ	$\nabla^2\rho$	V	G	K
C70 - H71	2.035736	0.290913	-1.065808	-0.326021	0.029785	0.296237
C70 - H72	2.050395	0.283322	-0.995352	-0.317747	0.034454	0.283292
C70 - N118	2.741458	0.265046	-0.688423	-0.438119	0.133006	0.305112
C70 - N120	2.736497	0.269615	-0.7042	-0.433294	0.128622	0.304672
C73 - C77	2.942297	0.243197	-0.602583	-0.256184	0.052769	0.203415
C73 - H74	2.045947	0.286307	-1.019476	-0.32076	0.032946	0.287815
C73 - N119	2.719829	0.280252	-0.745972	-0.440361	0.126934	0.313427
C73 - N120	2.771863	0.264352	-0.638722	-0.38873	0.114525	0.274205
C75 - N119	2.62523	0.311739	-0.941154	-0.566159	0.165435	0.400724
C75 - N121	2.633975	0.308387	-0.91175	-0.551194	0.161628	0.389566
C75 - O141	2.298134	0.419112	-0.367375	-1.432482	0.670319	0.762163
C76 - N120	2.636426	0.308022	-0.908024	-0.546339	0.159666	0.386672
C76 - N122	2.618059	0.314019	-0.954494	-0.576678	0.169027	0.407651
C76 - O142	2.301692	0.417575	-0.387652	-1.420101	0.661594	0.758507
C77 - H78	2.04608	0.28631	-1.019505	-0.320699	0.032911	0.287788
C77 - N121	2.760709	0.268127	-0.661647	-0.398549	0.116569	0.28198
C77 - N122	2.729084	0.277031	-0.726752	-0.43129	0.124801	0.306489
C79 - H80	2.036679	0.290627	-1.064741	-0.325427	0.029621	0.295806
C79 - H81	2.053086	0.282049	-0.985771	-0.316025	0.034791	0.281234
C79 - N121	2.733553	0.269474	-0.709193	-0.441281	0.131992	0.30929
C79 - N123	2.746356	0.265641	-0.682753	-0.424675	0.126993	0.297681
C82 - H83	2.035123	0.291207	-1.068845	-0.326235	0.029512	0.296723
C82 - H84	2.043279	0.286038	-1.016248	-0.321941	0.033939	0.288001
C82 - N122	2.747662	0.263271	-0.673182	-0.428795	0.13025	0.298545
C82 - N124	2.735246	0.268089	-0.700317	-0.439947	0.132434	0.307513

Atoms	distance (Å)	ρ	$\nabla^2\rho$	V	G	K
C85 - C89	2.949373	0.240825	-0.590301	-0.252144	0.052284	0.199859
C85 - H86	2.047343	0.285863	-1.015013	-0.320385	0.033316	0.287069
C85 - N123	2.757264	0.269685	-0.670694	-0.402483	0.117405	0.285078
C85 - N124	2.726448	0.278135	-0.732292	-0.433957	0.125442	0.308515
C87 - N123	2.641237	0.306974	-0.901418	-0.537296	0.155971	0.381326
C87 - N125	2.616446	0.314276	-0.95663	-0.578734	0.169788	0.408946
C87 - O143	2.30559	0.415544	-0.399645	-1.40726	0.653674	0.753586
C88 - N124	2.630435	0.308828	-0.919399	-0.557725	0.163938	0.393788
C88 - N126	2.635945	0.307584	-0.907257	-0.54742	0.160303	0.387117
C88 - O144	2.294092	0.421119	-0.348773	-1.446647	0.679727	0.76692
C89 - H90	2.047602	0.285681	-1.013994	-0.320056	0.033279	0.286777
C89 - N125	2.728596	0.277335	-0.728681	-0.433609	0.125719	0.30789
C89 - N126	2.750935	0.271491	-0.681568	-0.407358	0.118483	0.288875
C91 - H92	2.052374	0.282571	-0.98972	-0.316728	0.034649	0.282079
C91 - H93	2.035422	0.291166	-1.068866	-0.32615	0.029467	0.296683
C91 - N125	2.734371	0.267134	-0.702582	-0.445824	0.135089	0.310734
C91 - N127	2.74233	0.268179	-0.694915	-0.426551	0.126411	0.30014
C94 - H95	2.035282	0.291144	-1.068523	-0.32617	0.02952	0.29665
C94 - H96	2.049563	0.283697	-0.998183	-0.318373	0.034414	0.283959
C94 - N126	2.742842	0.267032	-0.690038	-0.428405	0.127948	0.300457
C94 - N128	2.733462	0.267766	-0.70591	-0.446208	0.134865	0.311343
Cu145 - N146	3.820193	0.089008	0.295563	-0.128514	0.101202	0.027312
Cu145 - N147	3.846796	0.08552	0.293665	-0.123977	0.098697	0.025281
Cu145 - N148	3.860326	0.084279	0.28772	-0.121054	0.096492	0.024562
Cu145 - N149	3.809586	0.090048	0.300442	-0.130999	0.103054	0.027944

Atoms	distance (Å)	ρ	$\nabla^2\rho$	V	G	K
N104 - H160	6.255802	0.002905	0.010882	-0.001255	0.001988	-0.000733
N105 - H161	5.208943	0.006769	0.022266	-0.003418	0.004492	-0.001074
N107 - H161	4.899287	0.008886	0.027673	-0.004461	0.005689	-0.001229
N111 - H182	6.983734	0.000883	0.002865	-0.000318	0.000517	-0.000199
N115 - H175	7.509248	0.000679	0.002624	-0.000225	0.000441	-0.000215
N116 - H175	7.294176	0.000792	0.00261	-0.000245	0.000449	-0.000204
N120 - H152	5.964181	0.003633	0.013122	-0.00164	0.00246	-0.00082
N121 - H151	4.917885	0.00909	0.030195	-0.004823	0.006186	-0.001363
N123 - H151	5.016102	0.007729	0.02619	-0.004081	0.005314	-0.001233
N126 - H155	6.343899	0.002349	0.009072	-0.000998	0.001633	-0.000635
N146 - C156	2.820612	0.247168	-0.559501	-0.351387	0.105756	0.245631
N146 - C162	2.818334	0.245772	-0.562268	-0.358483	0.108958	0.249525
N146 - H181	1.915302	0.327088	-1.634535	-0.50743	0.049398	0.458032
N147 - C159	2.823639	0.24594	-0.554691	-0.34998	0.105654	0.244326
N147 - C171	2.824109	0.243533	-0.552017	-0.356194	0.109095	0.247099
N147 - H182	1.901943	0.334109	-1.585176	-0.503808	0.053757	0.450051
N148 - C150	2.821592	0.246924	-0.559424	-0.35075	0.105447	0.245303
N148 - C174	2.825347	0.24381	-0.551857	-0.353104	0.10757	0.245534
N148 - H180	1.902887	0.333642	-1.607142	-0.506525	0.05237	0.454155
N149 - C153	2.816056	0.249029	-0.568468	-0.353672	0.105777	0.247895
N149 - C168	2.814934	0.246262	-0.566679	-0.363169	0.11075	0.252419
N149 - H183	1.91659	0.326383	-1.631392	-0.506769	0.049461	0.457309
O129 - H172	8.761546	0.000109	0.000402	-0.000025	0.000063	-0.000038
O130 - H155	5.733941	0.003227	0.012004	-0.001481	0.002241	-0.00076
O130 - H169	4.465112	0.011334	0.044487	-0.006812	0.008967	-0.002155

Atoms	distance (Å)	ρ	$\nabla^2\rho$	V	G	K
O132 - H181	3.768747	0.022293	0.084978	-0.016277	0.018761	-0.002484
O133 - H173	5.425727	0.003913	0.014349	-0.001858	0.002722	-0.000865
O134 - H158	4.360797	0.01251	0.048066	-0.007297	0.009657	-0.00236
O134 - O136	6.22481	0.004594	0.018256	-0.002232	0.003398	-0.001166
O135 - H173	4.997733	0.006794	0.02672	-0.00373	0.005205	-0.001475
O136 - H157	6.779812	0.003073	0.013479	-0.001515	0.002442	-0.000927
O137 - H179	6.518156	0.001122	0.004089	-0.000443	0.000733	-0.00029
O138 - H157	4.777051	0.007973	0.028904	-0.004183	0.005705	-0.001521
O138 - H164	4.648465	0.009106	0.035421	-0.005186	0.007021	-0.001835
O140 - H152	5.737436	0.004032	0.015127	-0.002068	0.002925	-0.000857
O140 - H183	3.726164	0.023647	0.088549	-0.017664	0.019901	-0.002237
O141 - H176	5.099512	0.00521	0.018992	-0.00257	0.003659	-0.001089
O142 - H154	4.439284	0.011979	0.045492	-0.006884	0.009128	-0.002245
O143 - H180	5.384916	0.004049	0.01549	-0.001946	0.002909	-0.000963
O144 - H154	6.214544	0.002792	0.011495	-0.001354	0.002114	-0.00076
